# Comparative Genomic Analysis of *slc39a12*/ZIP12: Insight into a Zinc Transporter Required for Vertebrate Nervous System Development

**DOI:** 10.1371/journal.pone.0111535

**Published:** 2014-11-06

**Authors:** Winyoo Chowanadisai

**Affiliations:** Department of Nutrition, University of California Davis, Davis, California, United States of America; University of Colorado, Boulder, United States of America

## Abstract

The zinc transporter ZIP12, which is encoded by the gene *slc39a12*, has previously been shown to be important for neuronal differentiation in mouse Neuro-2a neuroblastoma cells and primary mouse neurons and necessary for neurulation during *Xenopus tropicalis* embryogenesis. However, relatively little is known about the biochemical properties, cellular regulation, or the physiological role of this gene. The hypothesis that ZIP12 is a zinc transporter important for nervous system function and development guided a comparative genetics approach to uncover the presence of ZIP12 in various genomes and identify conserved sequences and expression patterns associated with ZIP12. Ortholog detection of *slc39a12* was conducted with reciprocal BLAST hits with the amino acid sequence of human ZIP12 in comparison to the human paralog ZIP4 and conserved local synteny between genomes. ZIP12 is present in the genomes of almost all vertebrates examined, from humans and other mammals to most teleost fish. However, ZIP12 appears to be absent from the zebrafish genome. The discrimination of ZIP12 compared to ZIP4 was unsuccessful or inconclusive in other invertebrate chordates and deuterostomes. Splice variation, due to the inclusion or exclusion of a conserved exon, is present in humans, rats, and cows and likely has biological significance. ZIP12 also possesses many putative di-leucine and tyrosine motifs often associated with intracellular trafficking, which may control cellular zinc uptake activity through the localization of ZIP12 within the cell. These findings highlight multiple aspects of ZIP12 at the biochemical, cellular, and physiological levels with likely biological significance. ZIP12 appears to have conserved function as a zinc uptake transporter in vertebrate nervous system development. Consequently, the role of ZIP12 may be an important link to reported congenital malformations in numerous animal models and humans that are caused by zinc deficiency.

## Introduction

Zinc is required for enzyme catalysis, cell signaling, and DNA repair by all organisms and is vital for growth and development and multiple physiological processes including immune and brain function [1]–[3]. During pregnancy, maternal zinc deficiency can increase the frequency of congenital malformations across many animal species [4]. Symptoms of zinc deficiency in humans include weight loss, severe dermatitis, slow wound healing, male hypogonadism, and reduced immune function [5]. Zinc deficient experimental diets in laboratory animals [6] or plant protein-based feed with low zinc bioavailability in livestock [7], [8] lead to similar symptoms such as impaired growth, development, fertility, and epidermal health.

Members of the solute carrier 39 (SLC39) gene family encode zinc transport proteins that are critical mechanisms for maintaining zinc homeostasis across a wide range of species [9]. The SLC39 family, with similarity to iron transporter IRT1 [10], is present in *Saccharomyces cerevisiae* yeast [11], *Arabidopsis* plants [12], invertebrates [13], and vertebrates [14] including humans [15], [16]. The phenotypic similarities of zinc deficiency across different mammals due to impaired intestinal zinc transport caused by spontaneous or targeted mutations of *slc39a4* (ZIP4) in humans [17], [18], mice [19], and cows [20] demonstrates how physiological functions for SLC39 members may be conserved across species.

The zinc transporter ZIP12 encoded by the *slc39a12* gene was uncovered by analyses of gene expression across different human tissues [21]. ZIP12, which highly expressed in the brain, is required for multiple aspects of neuronal differentiation including cAMP response element binding protein (CREB) phosphorylation and activity, neurite outgrowth, and microtubule polymerization and stability [21]. ZIP12 is also necessary for embryogenesis because inactivation of ZIP12 by antisense morpholino knockdown halted neural tube closure in *Xenopus tropicalis*, resulting in arrested development and lethality during neurulation [21].

Comparative genomics can be useful for determining the functions of genes in varied and assorted contexts. The general assumption of comparative genomics is the conservation of genomic sequence due to evolutionary constraints that imply some kind of biological importance or function [22] encoded by the conserved sequence. Comparative genomics may predict the physiological role of genes due to the relationship between tissue expression profiles and biological function [23]. Models and approaches based upon comparative genomics have been used to determine possible functions for genes and their protein products at different levels. For example, comparative genomics can be used to identify possible interaction partners [24], protein folding and structure [25], and the evolution of protein phosphorylation sites [26]. Comparative genomics may be able to identify genomic differences between organisms that confer physiological differences and species identity [22]. For example, nonsynonymous changes in the human *FOXP2* gene from non-human primates are hypothesized to be responsible for language development [27].

Because ZIP12 appears to be conserved and is highly expressed in the brain across humans, mice, and frogs [21], a comparative analysis of the *slc39a12* gene across multiple species and the SLC39 gene family was conducted to uncover information about this transporter. These analyses indicate that there are conserved elements in the *slc39a12* gene across various organisms that likely contribute towards important biochemical, physiological, and developmental functions for this zinc transporter. The presence of *slc39a12* in nearly all vertebrate genomes examined and a possible lack of *slc39a12* in other invertebrate organisms may indicate an association between ZIP12 and neurulation during vertebrate embryogenesis.

## Materials and Methods

### Sequence analyses, alignment, and phylogenetic tree

Nucleotide and amino acid sequences (Table 1) for *slc39a12* were obtained from annotated entries, BLASTP or TBLASTN using human *slc39a12* amino acid sequence, in Xenbase (for *Xenopus laevis*, 6.0 scaffolds) [28], Ensembl (for *Ciona intestinalis* and *Petromyzon marinus*) [29], Joint Genome Institute (for *Branchiostoma floridae*), or National Center for Biotechnology Information (NCBI) (all other organisms) [30]. UCSC Genome Browser, PhyloP, and PhastCons were used to display broad phylogenetic alignments and sequence conservation of *slc39a12* in vertebrates [31]. BLASTP was used to compare ZIP12 amino acid sequences of various organisms to both human ZIP12 and ZIP4. Additional information about *slc39a12* in *Xenopus laevis* was also obtained from daudin.icmb.utexas.edu. The amino acid sequence of ZIP12 from various organisms (see Table 1) was aligned by ClustalW [32] using Bioedit software version 7.0.9.0 [33]. The phylogenetic tree was drawn using TreeDyn [34] using Phylogeny.fr [35]. Because the existence of an exon, which is included in the long isoform, could not be confirmed in all organisms tested, the phylogenetic tree was drawn using only the putative short isoforms of ZIP12 from each organism. EST representations of *slc39a12* in the nervous system (brain, eye, spinal cord) and other tissues of different organisms were accomplished using NCBI Unigene [30]. Bioedit was used to calculate the nucleotide composition of the 5′ and 3′ untranslated and upstream regions.

**Table 1:** Orthologs of ZIP12 (*slc39a12*) are more similar to human ZIP12 than paralog ZIP4.

Animal	Species	Taxonomic Category	NCBI Gene ID	NCBI Accession DNA	NCBI Accession Protein	Human ZIP12	Human ZIP4
Human	*Homo sapiens*	Mammalia-Primate	221074	NM_001145195, NM_152725	NP_001138667, NP_689938	100%	31%
Chimpanzee	*Pan troglodytes*	Mammalia-Primate	739440	XM_001153573, XM_001153510	XP_001153573, XP_001153510	99%	36%
Rhesus monkey	*Macaca mulatta*	Mammalia-Primate	701128	XM_001092136	XP_001092136	97%	37%
Bushbaby	*Otolemur garnettii*	Mammalia-Primate	100948394	XM_003786777, XM_003786778	XP_003786825, XP_003786826	86%	36%
Rat	*Rattus norvegicus*	Mammalia-Rodent	291328	NM_001106124	NP_001099594	78%	37%
Mouse	*Mus musculus*	Mammalia-Rodent	277468	NM_001012305	NP_001012305	77%	38%
Guinea pig	*Cavia porcellua*	Mammalia-Rodent	100719835	XM_003462762, XM_003462763	XP_003462810, XP_003462811	84%	32%
European rabbit	*Oryctolagus cuniculus*	Mammalia-Other	100346557	XM_002717411, XM_002717412	XP_002717457, XP_002717458	83%	32%
Domestic dog	*Canis lupus familiaris*	Mammalia-Other	477989	XM_843257, XM_535173	XP_848350, XP_535173	84%	31%
Feral pig	*Sus scrofa*	Mammalia-Other	100523814	XM_003130728, XM_003130727	XP_003130776, XP_003130775	83%	33%
Horse	*Equus caballus*	Mammalia-Other	100056009	XM_003364356, XM_001497406	XP_003364404, XP_001497456	86%	38%
Cow	*Bos taurus*	Mammalia-Other	527210	NM_001076878	NP_001070346	84%	36%
Elephant	*Loxodonta africana*	Mammalia-Other	100671276	XM_003410560, XM_003410561	XP_003410608, XP_003410609	80%	37%
Opossum	*Monodelphis domestica*	Mammalia-Other	100026757	XM_001377227	XP_001377264	72%	34%
Platypus	*Ornithorhynchus anatinus*	Mammalia-Other	100078275	XM_001509297	XP_001509347	59%	36%
Chicken	*Gallus gallus*	Aves (Birds)	420514	XM_004939251	XP_004939308	62%	34%
Anole lizard	*Anolis carolinensis*	Reptilia	100552790	XM_003222078	XP_003222126	51%	40%
Western clawed frog	*Xenopus tropicalis*	Amphibia	100493177	XM_002939000	XP_002939046	51%	31%
African clawed frog	*Xenopus laevis*	Amphibia	JGI Xenla 6.0 Scaffold 172106	not available	not available	51%	37%
Nile tilapia	*Oreochromis niloticus*	Actinopterygii (Bony Fish)	100695101	XM_003439349	XP_003439397	50%	33%
European seabass	*Dicentrarchus labrax*	Actinopterygii (Bony Fish)	FQ310508 (chromosome)	(chromosome)	CBN82065	49%	34%
Spotted green pufferfish	*Tetraodon nigroviridis*	Actinopterygii (Bony Fish)	CAAE01015017 (chromosome)	(chromosome)	CAG10206	48%	37%
Japanese medaka	*Oryzias latipes*	Actinopterygii (Bony Fish)	NC_019878	XM_004081302	XP_004081350	50%	33%
Zebrafish	*Danio rerio*	Actinopterygii (Bony Fish)	NC_007135, NC_007113, NC_007118	not found	not found	N/A	N/A
Sea squirt	*Ciona intestinalis*	Ascidiacea (Tunicate)	100178452; Chr 8: 148339 to 149331	XM_002121310	XP_002121346	39%	40%
Sea lamprey	*Petromyzon marinus*	Agnatha	GL481648	ENSPMAT00000007608	N/A	47%	37%
Lancelet	*Branchiostoma floridae*	Cephalochordata (Amphioxus)	Scaffolds 92, 119, 158	not found	not found	N/A	N/A
Purple sea urchin	*Strongylocentrotus purpuratus*	Echinodermata	NW_003578262, NW_003577087	not found	not found	N/A	N/A
Fruit fly	*Drosophila melanogaster*	Insecta	NC_004354, NT_033779, NT_037436	not found	not found	N/A	N/A
Roundworm	*Caenorhabditis elegans*	Chromadorea (Nematode)	NC_003279	not found	not found	N/A	N/A

For taxonomic category, the class of each organism is provided. In cases where a NCBI Gene or Ensembl accession number is not available, an accession number for genomic sequences or information about the scaffold number is provided. Not found indicates that ZIP12 could not be detected in that organism, and not available indicates that ZIP12 is present in the organism, but that an accession number has not been assigned. Similarity of orthologs to human ZIP12 (NP_001138667, 100% for reference) and human ZIP4 determined by BLAST (one half of reciprocal BLAST, listed organism as query for human genome) and percent identities in amino acids. N/A (not applicable) is listed if no ortholog for ZIP12 can be detected in the organism genome. Additional entries indicate possible splice variation.

### Sequence motif scanning

Possible transcription factor binding sites were scanned using TRANSFAC (Match) Matrix Search for Transcription Factor Binding Sites [36]. Eukaryotic Linear Motif [37] was used to search for possible dileucine and tyrosine motifs associated with intracellular sorting and localization, as described previously by Huang and Kirschke for ZIP1 [38]. Positive matches for transcription factor binding sites and dileucine and tyrosine motifs were further examined for conservation across species in aligned amino acids sequences.

### Synteny analyses

Local synteny for *slc39a12* and neighboring genes (*cubn, vim, stam, mrc1, cacnb2, nsun6*, and *arl5b*) in most vertebrate organisms was discovered using NCBI Homologene [30]. Xenbase [28] was used for synteny analysis of *slc39a12* in *Xenopus tropicalis*. Searches for *slc39a12* and nearby genes in the genomes of zebrafish, *Drosophila*, *C. elegans*, and *Ciona intestinalis* were conducted using TBLASTN using translated protein versions of *cubn, vim, stam, mrc1, cacnb2, nsun6*, and *arl5b* in either tilapia or *Xenopus tropicalis* [30].

### Detection of splice variants of ZIP12 in different species

Bioinformatic searches for exon 9, which is not present in annotated entries for *slc39a12* in the chicken, cow, opossum, or platypus genomes, was detected by TBLASTN using a translated sequence (GLXLVNXHVGHXHHLXLNXELXDQXXXGKSXSTIQL) in exon 9 completely conserved across humans and mice.

Total brain RNA of human, cow, rat, and mouse origin was obtained from Zyagen (San Diego, CA). cDNA was reverse-transcribed from total RNA as described previously [21], using either a polyT primer or a ZIP12-specific primer (human and cow: acttatattttaatattttg; rat: atgtgaacatataaattcat; mouse: tgagtcatttcaggaagc). The different splice variants were detected using primers that flank exon 9, which is present in the long isoform and absent in the short isoform (Human- Fwd: acgctctgctccaccttatccctca, Rev: aaattatgcaggctgtccccaacca; Cow- Fwd: acagctgcgaggagaactacaggctca, Rev: ggttttgcattttctgttgggggtgtt; Rat- Fwd: cgaaagccaaagtcctatttggaagctg, Rev: gagcacagcaaagtctcccatttcatgt; Mouse- Fwd: taaccttgggctccatgcttgggacag, Rev: ggctggcacattgcctatgggtagcac) and the following PCR conditions: 94°C, 1 min; 37 cycles: 94°C, 30 s, 68°C, 1 min with Platinum Hifi Taq (Invitrogen, Carlsbad, CA). PCR products and DNA molecular weight markers (1 kb Trackit or 1kb+, Invitrogen) were separated by agarose gel electrophoresis and viewed as described previously [21]. From accompanying gels not exposed to ultraviolet light, bands corresponding to the different isoforms were excised (Qiagen, Hilden, Germany) and confirmed by DNA sequencing.

### Detection of *slc39a12* and *igf1* genes by PCR

Comparisons of genomic sequences for *slc39a12* of Japanese medaka, Nile tilapia, and European seabass by bl2seq were used to design PCR primers with some degeneracy. As a positive control, PCR primers were also designed for the *igf1* (insulin growth factor-1) gene present in zebrafish (NC_007115) using similar comparisons between Japanese medaka, Nile tilapia, and European seabass. This region of *igf1* was previously identified by Faircloth et al. [39] as containing an ultraconserved element among ray-finned fish (Node 267).

Genomic DNA was isolated from fish carcasses purchased at grocery stores (Nile tilapia and European seabass) using the QIAamp DNA Mini Kit (Qiagen). Zebrafish and Japanese medaka genomic DNAs were kindly provided as a gift by Bruce Draper (University of California-Davis) and Swee Teh (University of California-Davis). Using 100 ng of genomic DNA as a template, the *slc39a12* and *igf1* genes were amplified by PCR using the following primers (*slc39a12*- Fwd: ccantcanctggngganatt, Rev: attnccnagcaactgntga; *igf1*- Fwd: cccagctgtttcctgttgaa, Rev: ttccnactttgttccattgc; degeneracy underlined) and conditions: 94°C, 1 min; 40 cycles: 94°C, 20 s, 55°C, 20 s, 72°C, 40 s with GoTaq (Promega, Madison, WI), and PCR products were viewed as described above.

## Results and Discussion

**Figure 1:**
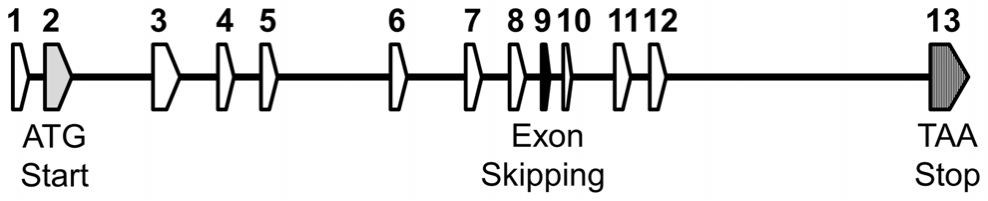
Human *slc39a12* gene structure. Exon-intron structure drawn to approximate scale. Exon 2 (shaded gray) contains the translation start codon. Exon 9 (shaded black) contains a variable exon depending on splice variation that leads to exon inclusion or exclusion. Exon 13 (striped) contains the stop codon (ochre). The exon structure (number of exons, relative exon size) of *slc39a12* is conserved in mice and *Xenopus tropicalis*.

**Figure 2:**
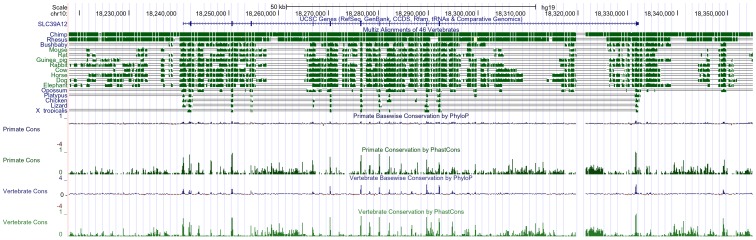
Phylogenetic alignment and nucleotide sequences of *slc39a12* show conservation across vertebrates. Human *slc39a12* gene structure is indicated. Multiz phylogenetic alignment of *slc39a12* orthologs in 16 vertebrate genomes show conservation in exons and some extra-exonic regions. Primate and vertebrate exonic and intronic regions of conservation are indicated by peaks following analysis by PhyloP and PhastCons. Scale bar at top indicates 50 kb.

**Figure 3:**
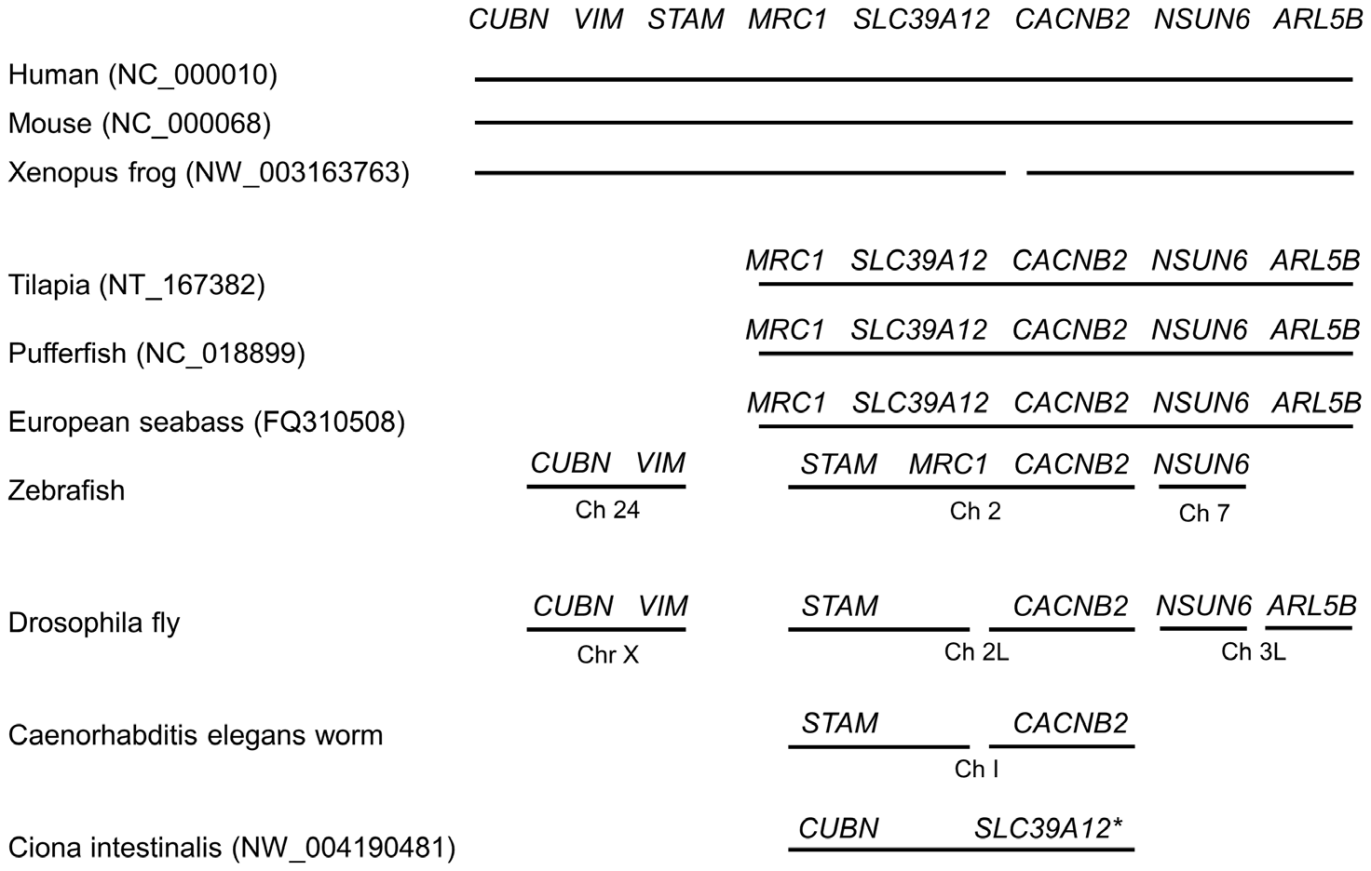
Synteny of *slc39a12* is preserved across nearly all vertebrates examined. *Xenopus* refers to *tropicalis* species of frog. NCBI accession numbers are indicated in parentheses where *slc39a12* is present. Gaps between solid lines within the same chromosome indicate that genes may be distant from each other. Chromosome number (Ch) is noted to indicate chromosome location of genes. The asterisk indicates that the putative *slc39a12* gene in *Caenorhabditis elegans* could not be confirmed using reciprocal BLAST hits.

**Figure 4:**
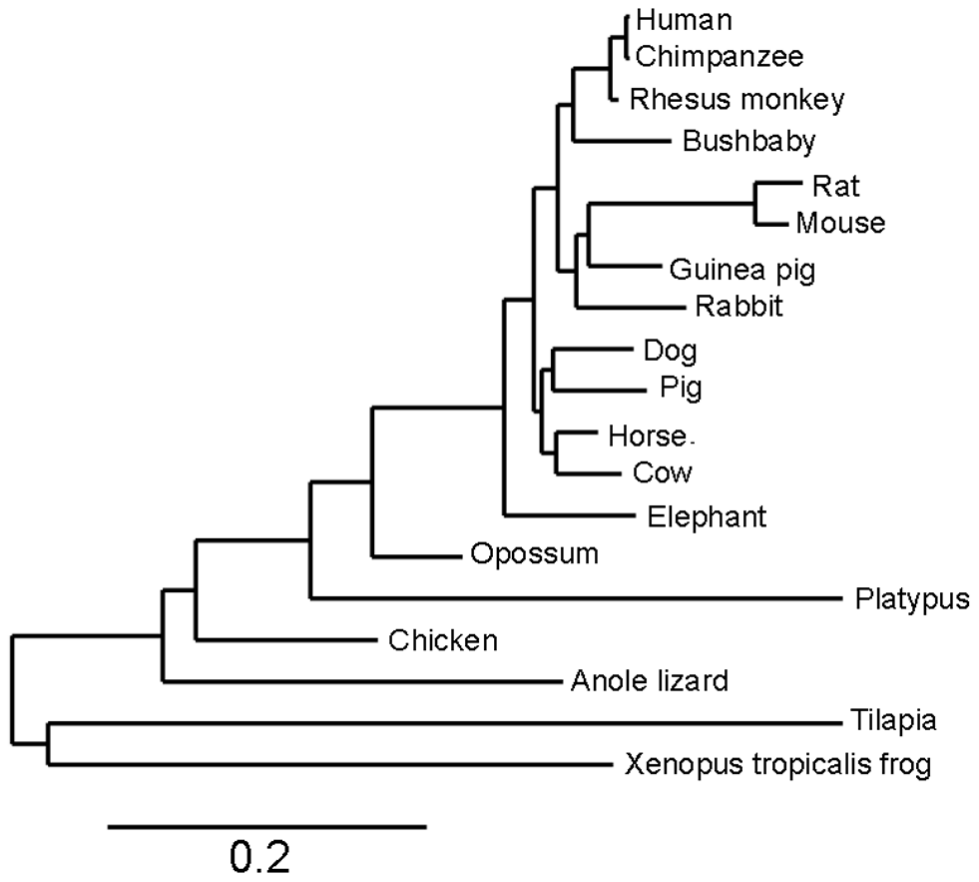
Phylogenetic tree based upon ZIP12 amino acid sequences in different species. Common names of organisms are listed. Scale bar indicates 0.2 amino acid substitutions per site.

The human *slc39a12* gene spans 13 exons across 91.4 kilobases (kb) on chromosome 10p12.33 (Figure 1, Figure 2). Orthologs to the human *slc39a12* gene (Table 1) were identified by combinations of Homologene searches, reciprocal BLAST hits [40], and local synteny preservation [41]. Identified orthologs of ZIP12 shared amino acid identities with the human ZIP12 that correlated with the relatedness of the organisms to humans, ranging from 86 to 99 percent for non-human primates to 48 to 50 percent in fish (Table 1). In contrast, the amino acid identities of the ZIP12 orthologs to human ZIP4 ranged between 31 to 40 percent without any correlation to relatedness with humans (Table 1). Because there are many common elements between members of the SLC39 gene family, particularly between ZIP12 and ZIP4 [9], [42], it can be difficult to distinguish orthologs of ZIP12 from SLC39 paralogs solely based upon sequence similarity such as BLAST. Local synteny between ortholog candidates is extremely useful in confirming reciprocal BLAST hits [41], especially in cases of gene families with large numbers of paralogs, such as the case with the solute carrier (SLC) gene families [43], [44]. In general, synteny was observed across vertebrates (Figure 3, Figure S1), but possible disruptions of the syntenic block in more distant organisms corresponded with reduced relatedness to humans, possibly due to genomic rearrangements during evolution [45]. As with the case with most orthologous genes in vertebrates [46], the coding exon structure of *slc39a12*, including number of exons and exon size, is conserved across humans, mice, and *Xenopus tropicalis* (data not shown). An alignment of human ZIP12 with other related ZIP genes (Figure S2) and with other ZIP12 orthologs (Figure S3) shows that many C-terminus elements are conserved, especially those predicted to encode the transmembrane helices and zinc transport function, such as the HEXPHEGD motif that is present in the LIV-1 subfamily of ZIP transporters [47]–[49]. The phylogenetic tree (Figure 4) indicates that the relatedness of ZIP12 across different organisms is highly correlated with the relatedness of the whole genomes across organisms [50]–[52].

*Xenopus laevis* embryology has a long history as a developmental model that precedes the use of *Xenopus tropicalis* in biological research, but the availability of a genome sequence for *Xenopus tropicalis* supports its use in genetic applications [53]. The larger size of *Xenopus laevis* embryo relative to *Xenopus tropicalis* can facilitate micromanipulations, such as microdissections, explants, tissue transplants, and targeted microinjections at advanced cleavage stages [54], [55]. Although *Xenopus laevis* has a pseudotetraploid genome [56] with some gene duplication, only a single match for *slc39a12* in *Xenopus laevis* [Joint Genome Institute (JGI) scaffold 000029451: 376741-407094] was detected, and the expected protein shares 85% amino acid identity with ZIP12 in *Xenopus tropicalis*. However, because the *Xenopus laevis* genome is still being assembled [57], the possibility of another duplicate *slc39a12* gene in the *Xenopus laevis* genome cannot be ruled out. The combination of a single ZIP12 ortholog in *Xenopus laevis* and larger embryo size may ease the study of ZIP12 in this organism and could complement further studies of ZIP12 in *Xenopus tropicalis*.

In agreement with Feeney et al. [58], attempts using different strategies to detect the *slc39a12* gene in zebrafish were unsuccessful. The *slc39a12* gene is present in other teleost fish, including tilapia, European sea bass, and green spotted pufferfish (Figure 3). Local synteny near *slc39a12* is preserved across humans, mice, European sea bass, and green spotted pufferfish (Figure 3). In contrast, for *slc39a12* and genes such as *cacnb2* and *nsun6* that surround *slc39a12* in other species (Figure S1), the genomic arrangement is disrupted in zebrafish. Furthermore, the *slc39a12* gene could be detected in Japanese medaka, European sea bass, and tilapia by PCR using degenerate primers, but *slc39a12* was not detectable in zebrafish (Figure S4). Because ZIP12 is required for embryonic viability in *Xenopus* [21], it is unclear how neurulation in zebrafish [59] proceeds without the presence of ZIP12. One possibility is that *slc39a12* resides in a region of the zebrafish genome that is still being resolved [60], but the disruption of microsynteny in surrounding genes in zebrafish relative to other teleosts and inability to detect *slc39a12* by PCR in zebrafish do not support this explanation. Although there are morphological differences in neurulation between amphibians and teleosts [61], *slc39a12* is present in other fish species and the cellular mechanisms in neurulation are conserved between frogs and fish. A likely explanation is that the function of ZIP12 has been substituted with another SLC39 family member, possibly due to genome duplication [62]. Studies have shown that the zebrafish has retained many duplicated genes, whereas the pufferfish has lost many of the duplicated genes [63]. More research is required to resolve this issue and to provide an explanation for the apparent absence of the *slc39a12* gene in zebrafish.

The identification and comparison of genes in invertebrates and vertebrates can provide significant insight into the origins of genes and their role in development. Neurulation is limited to chordates, including *Ciona* [64] and *Petromyzon* [65], but *Drosophila* [66], *C. elegans* [67], and *Strongylocentrotus* [68] have nervous systems but do not undergo neural tube closure. Neurulation in *Branchiostoma* proceeds with some distinct differences compared to vertebrates [69]. Regulatory subfunctionalization, which occurs through changes in cis-regulatory domains of duplicated genes, is recognized as a possible mechanism for the development of paralogs following a presumed gene duplication event [70]. For zinc transporter genes of the SLC30 family, Gustin et al. [71] proposed that the retention of duplicated genes occurs through changes in the expression patterns leading to eventual neofunctionalization or subfunctionalization. To test for a role of ZIP12 in neurulation, searches for ZIP12 were conducted in invertebrates with no or limited aspects of neural tube closure during embryogenesis. If ZIP12 functions in a critical role during neural tube closure, then positive selection may apply to *slc39a12* in vertebrate genomes, whereas organisms that do not undergo neurulation may lack the evolutionary pressures that led to the emergence of *slc39a12* in vertebrates. The criteria used to successfully identify ZIP12 in vertebrates, including reciprocal BLAST, conserved synteny, and conserved HNFAD and HEIPHE motifs in predicted transmembrane helices (Figure S2), was used to detect ZIP12 in other organisms.

There are possible matches for ZIP12 in *Ciona intestinalis* and *Petromyzon marinus* with similarity to human ZIP12, but a *slc39a12* gene could not be conclusively defined in these genomes. The putative sea squirt and lamprey genes have the motifs HNFTD-HEIPHE and HNFAD-HEVPHE that are near-matches for conserved transmembrane helices of ZIP12 in vertebrates. Although BLAST searches using human and tilapia ZIP12 amino acid sequences identify putative ZIP12 genes in tunicates and sea lampreys, reciprocal BLAST searches were unable to distinguish the putative tunicate and sea lamprey *slc39a12* genes as orthologs distinct from *slc39a4*. The putative *Ciona slc39a12* gene is located within 20 kb of an ortholog for cubulin, a gene that neighbors *slc39a12* in many vertebrate species (Figure 3). The limited size of the genome scaffold and putative transcripts for ZIP12 available in the sea lamprey restricted the analysis of *slc39a12* and did not enable synteny analysis. Two intriguing questions are whether tunicates and lampreys contain *slc39a12* genes with a similar function to ZIP12 in vertebrate neurulation. Future experiments may shed light on the developmental regulation of zinc transport through functional characterization of putative zinc transporters of the SLC39 gene family, and both gain-of-function and loss-of-function approaches are possible in tunicate and lamprey embryos [71], [72].

Attempts to find ZIP12 in fruit flies, nematodes, sea urchins, and lancelets using BLAST were unsuccessful. Local synteny in *Drosophila, C. elegans, Strongylocentrotus,* and *Branchiostoma* was not conserved for the genes that neighbor *slc39a12* in vertebrates (Figure 3). ZIP12 is required for both neurulation during embryogenesis and neurite outgrowth in Neuro-2a cells and primary mouse neurons [21]. However, the inability to conclusively identify ZIP12 in these invertebrates, despite the presence of a nervous system, suggests a possible link to neural tube closure as opposed to later stages of nervous system development. It is possible that the origins of *slc39a12* come from the requirement for zinc transport during neurulation in vertebrates (and possibly chordates), and that a role of ZIP12 in vertebrate nervous system function evolved following neurulation.

There may be some plausible alternate explanations for the observed lack of *slc39a12* in invertebrates. For example, the inability to detect ZIP12 may be due to inadequate or overly stringent search criteria or incomplete genome coverage. It is possible that a similar ZIP transporter such as ZIP4 or other metal-permeable transporters may substitute for ZIP12 in other organisms. For example, a TRP channel mediates zinc transport and is critical for development in *Drosophila* [73], an organism in which ZIP12 was not detected. However, the lack of *slc39a12* in zebrafish, a teleost that undergoes neurulation [59], represents an important observation that conflicts with the putative association between ZIP12 and vertebrate neurulation. Functional characterization across various organisms such as mice, frogs, and other developmental model organisms may shed light about the role of ZIP12, other ZIP transporters, and zinc regulation during nervous system development.

**Figure 5:**
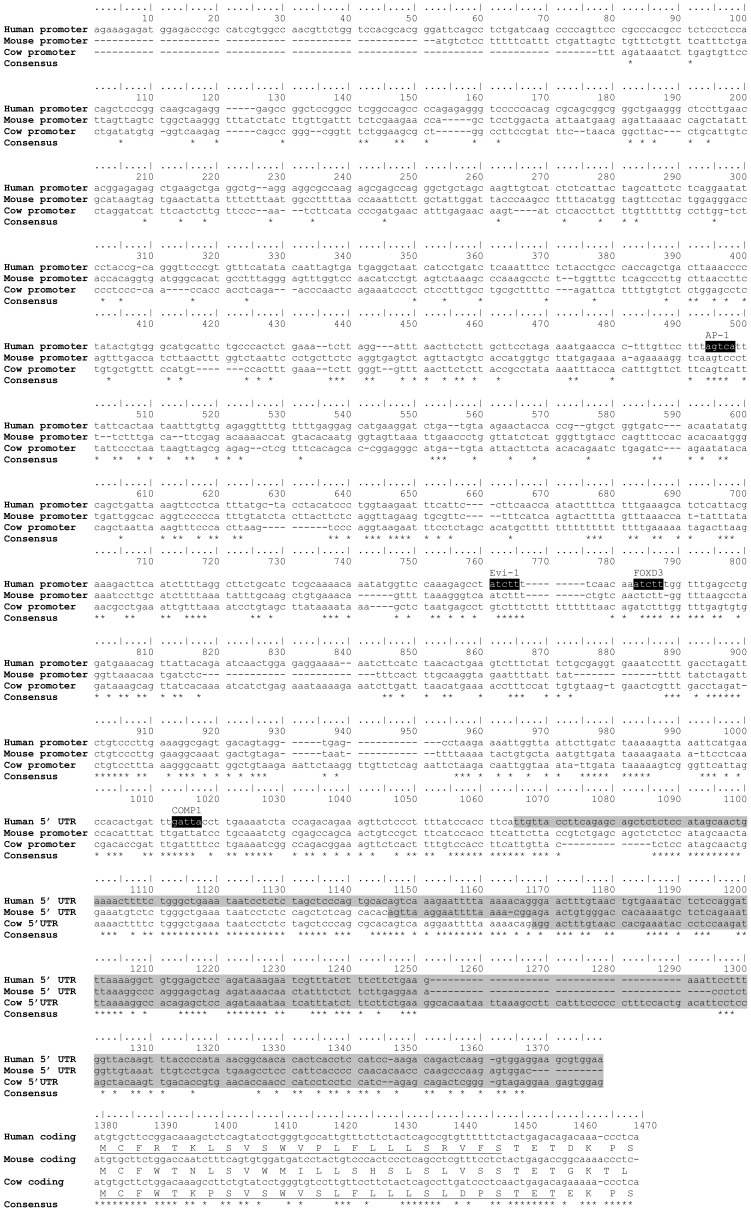
Alignment for 5′ UTR, first 90 bp of coding region, and the proximal promoter (1000 bp upstream of transcription start site) of human, mouse, and cow *slc39a12*. Lowercase and uppercase in sequences indicate nucleotide and amino acid sequences, respectively. Black shaded text indicates possible transcription factor binding sites in largely conserved regions. Gray shaded text indicates 5′ UTR. For coding regions, possible signal peptide is underlined. Asterisks indicate nucleotides conserved in all sequences following alignment.

Many of the predicted sequences for *slc39a12* (Table 1) were performed through computational analyses [30], and some sequences lack biological evidence such as cDNA support. Bioinformatic and computational analyses are extremely useful, especially for genome-wide annotation of genes, but there can be some discrepancies or unresolved regions [74]. Accordingly, some differences were detected between annotated versions of *slc39a12* solely derived from computation and other versions of *slc39a12* with additional bases including expressed sequence tags (ESTs) and other biological experimentation. Comparisons with other ZIP12 sequences showed that the rat ZIP12 protein sequence [GenBank: NP_001099594] was likely missing the N-terminus (Figure S3). As a result, the N-terminus of ZIP12 in this report was reconstituted from the translated sequences of a rat EST [GenBank: FM065041] and 12 nucleotides of the rat genome [GenBank: NW_047496 3955687-3955698] (Figure 4, Figure S3). The 5′ untranslated region (UTR) of the annotated cow ZIP12 [GenBank: NM_001076878] (Figure 5) is missing at least 60 bp, based upon EST data and the observation that the conserved exon-intron structure of human, mouse, and chicken *slc39a12* has the start codon in exon 2 (Figure 1, Figure 2). A comparison of the 5′ end of 2 EST clones [GenBank: EV626550 and EE901356] aligns to a separate exon in the cow genome [GenBank: AC_000170, 32666526-32666609]. The refinement of draft genome sequences and the correct annotation of gene sequences is important because genome sequences often provide the initial foundation for biological experimentation, particularly in reverse genetics.

ZIP12 is highly expressed in human, mouse, and *Xenopus tropicalis* brain tissue [21]. In support of this observation, the majority of expressed sequence tags (ESTs) matching ZIP12 is derived from brain or other nervous system tissues. Over-representation of ZIP12 in the brain occurred in almost all mammalian and avian species examined, including human, mouse, cow, pig, rhesus monkey, chicken, rat, and crab-eating macaque (Table 2). Only in dogs (Table 2) were ESTs not derived from brain tissue, and the pool of ESTs that match ZIP12 in dogs has few samples. This observation largely supports the notion that ZIP12 function in the nervous system is widely conserved across vertebrates. The conserved expression of ZIP12 in the brain across species is significant because the rate of evolution in tissue-specific genes appears to be faster and genetic disorders with Mendelian inheritance are more likely to be caused by mutations in tissue-specific genes [75]. Consistent with this observation, inhibition of ZIP12 function in mouse neurons and *Xenopus tropicalis* embryos leads to impaired neuronal differentiation and neural tube defects, respectively [21].

**Table 2:** Tissue origin of ESTs matching ZIP12 is predominantly brain and nervous system.

Organism	Unigene ID	ESTs from brain	3′ UTR A/U
Human (*Homo sapiens*)	193909	129/135 (95.6%)	A:32.9% U:40.3%
Mouse (*Mus musculus*)	271009	31/47 (66.0%)	A:31.8% U:41.8%
Cow (*Bos taurus*)	1209583	30/42 (71.4%)	A:33.3% U:41.2%
Pig (*Sus scrofa*)	3109541	13/14 (92.9%)	A:32.0% U:39.0%
Chicken (*Gallus gallus*)	1236550	5/8 (62.5%)	A:30.0% U:34.4%
Macaque (*Macaca fascicularis*)	2484482	5/5 (100.0%)	A:30.9% U:40.4%
Rat (*Rattus norvegicus*)	3092273, 1534920	4/5 (80.0%)	A:31.6% U:41.5%
Rhesus monkey (*Macaca mulatta*)	5927016	2/2 100.0%	A:31.2% U:40.3%
Dog (*Canis lupus familiaris*)	1193028	0/2 0%	A:31.5% U:38.8%

The Unigene ID number for each organism (common and scientific name) is listed. The proportion and percentage of ESTs from brain, out of the total number of ESTs matching ZIP12, are given. Data listed in order of sample size (total of ESTs) available from Unigene database. Brain includes all tissues for central nervous system, including brain, eye, and inner ear. Both ESTs matching ZIP12 from dog originate from heart tissue. The composition of adenine (A) and uracil (U) in the 3′ untranslated region (UTR) of *slc39a12* for each organism is listed.

**Figure 6:**
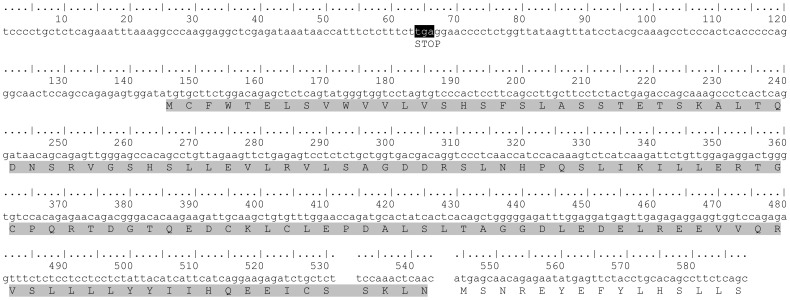
Rat *slc39a12* mRNA and translated protein sequences derived from EST and genome analyses. Full sequence was formed from rat EST [GenBank: FM065041], genome sequence [GenBank: NW_047496, nucleotides 3955687-3955698], and current annotated entry for rat *slc39a12* [GenBank: NM_001106124]. The additional N-terminus amino acid sequence is shaded gray. Stop codon (opal) upstream of putative start codon is shaded black.

There are possible mechanisms outside the coding sequence which may control the tissue-specific expression of *slc39a12* in vertebrates and are likely conserved. The 3′ untranslated region of ZIP12 in many species is adenine/uracil (A/U) rich (Table 2). The 3′ UTRs of many brain-specific genes are A/U rich [76]. There are proteins that can affect mRNA stability by binding to these A/U rich regions [77], which may account for the high expression of ZIP12 in the nervous system. An alignment of the 5′ UTR, the first 90 bp of the coding sequence, and the proximal promoter (1000 bp upstream of the transcription start site) (Figure 5) shows that there are areas of sequence conservation. A scan of the human proximal promoter combined with an alignment of the sequences with cow and mouse shows that there are putative transcription binding sites for AP-1, Evi-1, FoxD3, and COMP1 (Figure 6). It is possible that the N-terminus encodes a signal peptide, and the prediction software SignalP [78] indicates that there may be a cleavage site between amino acids 23–24 or 26–27 (Figure 5). In contrast to many genes important for neuronal development [79], the 5′ UTR and proximal promoter are not guanine/cytosine (GC) rich (GC content: human 44.8%; mouse 39.3%; cow 39.8%), and there was no difference in GC content between the 5′ UTR (41.8%) and proximal promoter (45.6%) of the human *slc39a12* gene. More analyses, possibly combined with biological experimentation, will be needed to determine if the 5′ UTR, 3′ UTR, or nearby upstream portions contribute towards the high expression of ZIP12 in the nervous system. As an example of how regulatory elements can control zinc uptake transporter expression, active metal-response elements have been identified in the 5′ UTR of zinc transporter genes in mice and zebrafish [80], [81]. Because there are possible conserved elements in the distal promoter and some intronic regions, as indicated by PhyloP and PhastCons (Figure 2), these areas may also contribute towards the distinct pattern of ZIP12 expression in the vertebrate nervous system and the possible regulatory subfunctionalization of ZIP12 and close paralogs. The tissue specificity of *slc39a12* in these species and previous findings showing enriched expression of *slc39a12* in vertebrate brains [21] support a role for ZIP12 in the central nervous system.

**Figure 7:**
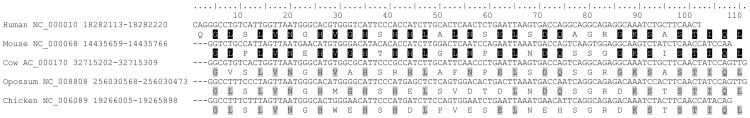
Histidine-rich exon 9 present in human and mouse ZIP12 is also present in cow, opossum, and chicken genomes. Organism common names are accompanied by accession numbers, corresponding nucleotides, and translated amino acid sequence. Amino acids conserved between humans and mice are shaded in black. Corresponding amino acids that are conserved in cow, opossum, and chicken are shaded in grey.

**Figure 8:**
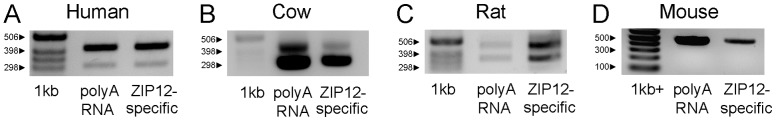
Splice variation of ZIP12 confirmed in the brain of multiple species. Different splice variants of ZIP12 are present in (A) humans, (B) cow, and (C) rat, but the short variant is not detectable in (D) mice. PCR was conducted on cDNA reverse-transcribed from polyadenylated or ZIP12-specific RNA using primers spanning the exon that is present and absent in the long and short isoform, respectively. The corresponding molecular weights of the DNA markers are indicated.

**Figure 9:**
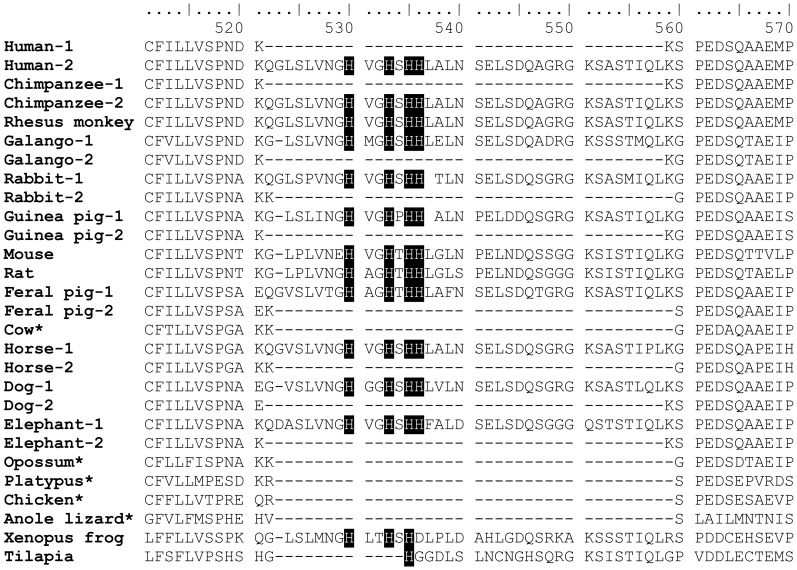
Amino acid alignment of ZIP12 demonstrates splice variation due to variable inclusion of a exon which contains a histidine-rich motif. Where indicated, -1 and -2 indicate annotated entries for splice variants of ZIP12 from inclusion or exclusion of exon 9. Shown alignment performed by ClustalW corresponds to amino acids 464–523 of the longer human ZIP12 variant (Human-2) [GenBank: NP_001138667]. Conserved histidine residues are shaded in black. Asterisks indicate organisms with a single annotated amino acid sequence for ZIP12 that lacks exon 9. Full sequence alignment is provided in Figure S3.

The alignment (Figure S3) uncovered 2 different isoforms of ZIP12 frequently detected across many species which correspond to the inclusion or exclusion of exon 9. Furthermore, this exon was present in at least two non-mammalian organisms, *Xenopus tropicalis* and tilapia (Figure S1), which supports an ancestral history for this exon in *slc39a12* that precedes the split with birds and mammals [82]. Although this exon is likely present in other mammalian organisms, the longer isoform has not been described previously in chicken, cow, opossum, or platypus. TBLASTN searches for exon 9 showed that the sequence is present in cow, chicken, and opossum genomes (Figure 7). This sequence was not detected in the platypus, but this may be due to gaps in the genomic sequence [GenBank: NW_001594582, 1525899-1525899, 1527878-1528642]. Reverse transcriptase-polymerase chain reaction (RT-PCR) was used to determine that both isoforms are expressed in humans, cows, and rats (Figure 8A-8C). However, the shorter isoform in mice (Figure 8D) could not be detected by RT-PCR despite repeated attempts with different primer sequences and PCR cycling conditions (data not shown). The reading frame in this exon appears to be conserved across species (111 bp in humans and 108 bp in other species, Figure 9). It is possible that the reading frame of this exon is conserved, so that inclusion or exclusion of this exon does not affect the downstream reading frame. The translated product of this exon is expected to increase the length of a cytoplasmic loop between transmembrane domains 3 and 4 of ZIP12 [21]. The transcript variation of ZIP12 is likely due to exon skipping, which is the most common form of splice variation [83]. The intron-exon structure flanking exon 9 is conserved across multiple vertebrate and mammalian species (Figure 2), which supports the notion that this variation has biological significance. Because this region of ZIP12 encodes a histidine-rich segment that is expected to lengthen a cytoplasmic loop [21], [48], this region could be important for post-translational regulation of ZIP12. In support of this possible function, ZIP4 contains a similarly located histidine-rich, cytoplasmic-facing motif that is sensitive to zinc and required for ubiquitin-mediated protein degradation in response to excess zinc [47]. Wide-scale global approaches have used comparative genomics to discover novel human exons that were previously unidentified because of weak or lacking cDNA support due to low transcript levels or restricted tissue specificity [84].

ZIP12 is present at both the plasma membrane and in intracellular compartments in a similar fashion to other SLC39 transporters [21], [38], [85], but it is unclear what mechanisms control the localization of ZIP12. Human ZIP12 has possible di-leucine motifs [86] at L94-L95 (EPDALLI), L116-L118 (QRVSLLL), and L255-L256 (ELDQLLL) and a possible tyrosine motif at Y120 (YYII) that are conserved in many mammalian species (Figure S3). Adaptor protein complexes recognize both di-leucine and tyrosine motifs and bind to transmembrane proteins to dynamically alter their cellular localization [86]. Human ZIP1 has a di-leucine motif that is important for endocytosis from the plasma membrane [38], [86], which likely controls zinc transport activity by withdrawing the zinc transporter from the plasma membrane [85] so that zinc can no longer be transported from the extracellular media by ZIP1. ZIP4 contains an ectodomain that is proteolytically cleaved during zinc deficiency, which could be due to a metalloprotease cleavage site [87]. The ectodomain of ZIP6 (LIV-1) can also be separated from its transmembrane-spanning C-terminus [88], [89]. Kambe and Andrews previously reported that the putative metalloproteinase site in ZIP4 is conserved at amino acids 338–341 (PGII) in human ZIP12 [87]. It is possible that these motifs that are conserved in ZIP12 and present in other SLC39 transporters are important for regulating the biochemical and cellular properties of this zinc transporter.

## Conclusion

The conservation of ZIP12 expression in the nervous system of vertebrates, combined with the previous report of the critical role of ZIP12 in *Xenopus* embryogenesis and mouse neuronal development supports the notion that zinc transport by ZIP12 is crucial for many vertebrate organisms. Zinc deficiency in numerous animal models is associated with congenital malformations and impaired development including neural tube defects [4], [6], [19]. Conserved gene co-expression analysis [23] and genotype-phenotype relationships (phenologs) across species [90] have been hypothesized by other researchers to predict candidate genes in human disease. These observations further strengthen the notion that *slc39a12* may be a candidate gene for neural tube defects or neurodevelopmental disorders [21].

The role of zinc in the evolution of embryogenesis is not currently well-studied, but the wide presence of ZIP12 in vertebrates and possibly chordates suggests a physiological importance of zinc transporters in these pathways. Cellular zinc homeostasis and free zinc availability is tightly regulated [91], even in bacteria, but there is emerging evidence shows that zinc may affect cell signaling [21], [49] and post-transcriptional mechanisms [13]. It is possible that the origins of zinc transporter functions in cell signaling originated from the role of related paralogs and their roles in zinc homeostasis and detoxification. Further investigation is required in order to determine the origins of ZIP12 in relation to the evolution of developmental processes and the likely significant role of ZIP12 in nervous system function in vertebrate organisms.

## Supporting Information

Figure S1 **Synteny of** ***slc39a12*** **is preserved across nearly all vertebrates examined.** NCBI accession numbers are indicated in parentheses. Solid lines indicate that neighboring genes on the same chromosome.
(TIF)

Figure S2 **Sequence alignments of different members of the SLC39 (ZIP) family.** The amino acid sequences (and NCBI accession numbers) are as follows: human ZIP12 [GenBank: NP_001138667] and ZIP1 [GenBank: NP_055252], *Saccharomyces cerevisiae* (yeast) ZRT1 [GenBank: NP_011259], *Arabidopsis ZIP2* [GenBank: NP_200760], and mouse ZIP4 [GenBank: NP_082340], ZIP6 [GenBank: NP_631882], and ZIP14 [GenBank: NP_001128623]. The consensus sequence is represented below the alignment, ranging from no (blank), low (.), medium (:), to high conservation (*). Conserved amino acids from the putative transmembrane domains 4 and 5 are indicated by gray shading.
(PDF)

Figure S3 **Sequence alignments of ZIP12 orthologs from different vertebrates.** NCBI accession numbers for amino acid sequences are provided in Table 1. The consensus sequence is represented below the alignment, ranging from no (blank), low (.), medium (:), to high conservation (*).
(PDF)

Figure S4 **The** ***slc39a12 gene*** **is detectable in Japanese medaka, Nile tilapia, and European seabass but not zebrafish.** The *slc39a12* and *igf1* genes were detected by PCR using genomic DNA from zebrafish, Japanese medaka, Nile tilapia, and European seabass and primers with degeneracy. In zebrafish, only the *igf1* gene was detected, whereas both *slc39a12* and *igf1* genes were detected in the other fish. The expected PCR product sizes for *slc39a12* and *igf1* are 106 bp and 130 bp, respectively.
(TIF)
