# Supplementary material for: Comparative Genomic Analysis of slc39a12/ZIP12: Insight into a Zinc Transporter Required for Vertebrate Nervous System Development
Source: PLoS One. 2014 Nov 6;9(11):e111535. doi: 10.1371/journal.pone.0111535 (PMC4222902; doi:10.1371/journal.pone.0111535)
Supplement: Figure S2 — Sequence alignments of different members of the SLC39 (ZIP) family. The amino acid sequences (and NCBI accession numbers) are as follows: human ZIP12 [GenBank: NP_001138667] and ZIP1 [GenBank: NP_055252], Saccharomyces cerevisiae (yeast) ZRT1 [GenBank: NP_011259], Arabidopsis ZIP2 [GenBank: NP_200760], and mouse ZIP4 [GenBank: NP_082340], ZIP6 [GenBank: NP_631882], and ZIP14 [GenBank: NP_001128623]. The consensus sequence is represented below the alignment, ranging from no (blank), low (.), medium (:), to high conservation (*). Conserved amino acids from the putative transmembrane domains 4 and 5 are indicated by gray shading. (PDF) [file pone.0111535.s002.pdf]

```
                 ....|....| ....|....| ....|....| ....|....| ....|....| ....|....| ....|....| ....|....| ....|....| ....|....|
                         10         20         30         40         50         60         70         80         90        100
Human ZIP12      MCFRTKLSVS WVPLFLLLSR VFSTETDKPS AQDSRSRGSS GQPADLLQVL SAGDHPPHNH SRSLIKTLLE KTGCPRRRNG MQGDCNLCFE PDALLLIAGG
Human ZIP1       ---------- ---------- ---------- ---------- ---------- ---------- ---------- ---------- ---------- ----------
Yeast ZRT1       ---------- ---------- ---------- ---------- ---------- ---------- ---------- ---------- ---------- ----------
Arabidopsis ZIP2 ---------- ---------- ---------- ---------- ---------- ---------- ---------- ---------- ---------- ----------
Mouse ZIP4       -MLPKSVTQG LVLALLVGTV AVARPRNLLS LLALG----- ---------- --QGALDRLE LDGLLNTLVA RVHCTDG--- ---PCEKCLS VENVLALGKP
Mouse ZIP6       -------MAT DLSVIMILTF ALWVTSPLHE LQSTAAFSQT TEKINSNWEP GVNVDLAVTM QRHHLQQLFY RYG------- ---------E NDSLSVEGFR
Mouse ZIP14       ---MKRLHPA LPSCLLLVLF GIWRTAPQTH ASS------- ---------- ---AGLPPLS ATSFLEDLMD RYG------- ---------K NDSLTLTQLK
Consensus

                 ....|....| ....|....| ....|....| ....|....| ....|....| ....|....| ....|....| ....|....| ....|....| ....|....|
                        110        120        130        140        150        160        170        180        190        200
Human ZIP12      NFEDQLREEV VQRVSLLLLY YIIHQEEICS SKLNMSNKEY KFYLHSLLSL RQDEDSSFLS QNETEDILAF TRQYFDTSQS QCMETKTLQK KSGIVSSEGA
Human ZIP1       ---------- ---------- ---------- ---------- ---------- ---------- ---------- ---------- ---------- ----------
Yeast ZRT1       ---------- ---------- ---------- ---------- ---------- ---------- ---------- ---------- ---------- ----------
Arabidopsis ZIP2 ---------- ---------- ---------- ---------- ---------- ---------- ---------- ---------- ---------- ----------
Mouse ZIP4       DKPQPAPESV LESR------ HIIYLSAAAA LYLNNPEKTC KDIQAGLLAS HVDDYLATLE SPEAMTLGLS QLLQKIEAHA ASQPTGEKTC VDLPQLLEEA
Mouse ZIP6       KLLQNIGIDK IKRV------ HIHHDHEHHA DHEHHSDHEH HSDHEHHSDH EHHSDHEHHS DHEHHSHRSH TVAGKNNRKA FCPDLDSDNS GKNPRTSLGK
Mouse ZIP14       SLLDHLHVGV GR-------- ---------- ---------- ---------- ---------- ---------- ---------- ---------- ----------
Consensus

                 ....|....| ....|....| ....|....| ....|....| ....|....| ....|....| ....|....| ....|....| ....|....| ....|....|
                        210        220        230        240        250        260        270        280        290        300
Human ZIP12      NESTLPQLAA MIITLSLQGV CLGQGNLPSP DYFTEYIFSS LNRTNTLRLS ELDQLLNTLW TRSTCIKNEK IHQFQRKQNN IITHDQDYSN FSSSMEKESE
Human ZIP1       ---------- ---------- ---------- ---------- ---------- ---------- ---------- ---------- ---------- ----------
Yeast ZRT1       ---------- ---------- ---------- ---------- ---------- ---------- ---------- ---------- ---------- ----------
Arabidopsis ZIP2 ---------- ---------- ---------- ---------- MALSSKTLKS TLFFLS---- ---------- ---------- ---------- ----------
Mouse ZIP4       EAAGVSKSAG LVLTALLDHV INGSCFQGLP SPQYFVDFVF RLHSSDPPNI TLHELENLMH HLGVGGEDHS DHDDHGDHAD HSHPDRKASH QDSELHTPHN
Mouse ZIP6       GSRPAEHMNG RRNIKESASS SEVTSAVYNA VSEGTRFVET IETPKPGRRT KDVNPSTPPS ITEKSRVGRL SRLARKKSNE SVSEPRKSFM YSRNTN----
Mouse ZIP14       ---------- ---------- ---------- --------DN VSQPKEGPR- ---------- ---------- ---------- ---------- ----------
Consensus

                 ....|....| ....|....| ....|....| ....|....| ....|....| ....|....| ....|....| ....|....| ....|....| ....|....|
                        310        320        330        340        350        360        370        380        390        400
Human ZIP12      DGPVSWDQTC FSARQLVEIF LQKGLSLISK EDFKQMSPGI IQQLLSCSCH LPK---DQQA KLPP---TTL EKYGYSTVAV TLLTLGSMLG TALVLFHSCE
Human ZIP1       -----MGPWG EPELLVWRPE AVASEPPVPV GLEVKLGALV LLLVLTLLCS LVP------- ---------- ---------- --ICVLRRPG ----ANHEGS
Yeast ZRT1       --------MS NVTTPWWKQW DPSEVTLADK TPDDVWKTCV LQGVYFGGNE YNG------- --------NL GARISSVFVI LFVSTFFTMF PLISTKVKRL
Arabidopsis ZIP2 -----IIFLC FSLILAHGGI DDGDEEEETN QPPPATGTTT VVNLRSKSLV LVK------- ---------- ---------I YCIIILFFST FLAGVSPYFY
Mouse ZIP4       SNSSVWDTLC LSAKDIMAVY GLSEEAGVSP QAWAQLTPAL VQQQLSGACS PYP------T IRIQDQLSQT ERYLYGSLAT LLICLCAVFG LLLLTCAKCS
Mouse ZIP6       ----DNIQEC FNTTKLLTSH GMSIQALLNA TEFNYLCPAI INQIDARACL IHT-ASEKKA EIPPKTYSLQ IAWLGGFIAI SIISFLSLLG -VILVPLMNR
Mouse ZIP14       -----NLSTC FSSGDLFAAH NLSERSQIGA SEFQEFCPTI LQQLDSQACT SENQKSEENE QTEEGKPSAI EVWGFGFLSV SLINLASLLG -VLVLPCTEK
Consensus                                             :                                                      .    ::

                 ....|....| ....|....| ....|....| ....|....| ....|....| ....|....| ....|....| ....|....| ....|....| ....|....|
                        410        420        430        440        450        460        470        480        490        500
Human ZIP12      ENYRLILQLF VGLAVGTLSG DALLHLIPQV LGLHKQEAPE FGHFHESK-- ---------- -------GHI WKLMGLIGGI HGFFLIEKCF ILLVSPNDK-
Human ZIP1       ASRQKALSLV SCFAGGVFLA TCLLDLLPDY LAAIDEALAA LHVTLQFP-- ---------- ---------- LQEFILAMGF FLVLVMEQIT LAYKE-----
Yeast ZRT1       RIPLYVYLFA KYFGSGVIVA TAFIHLMDPA YGAIGGTTCV GQTGNWG--- ---------- -------LYS WCPAIMLTSL TFTFLTDLFS SVWVERKYG-
Arabidopsis ZIP2 RWNESFLLLG TQFSGGIFLA TALIHFLSDA NETFRGLKHK EYP------- ---------- ---------- YAFMLAAAGY CLTMLADVAV AFVAAGSNN-
Mouse ZIP4       TATHYIMQTF LSLAVGALTG DALLHLIPKV LGLHTHGGEG HTHEEEVGVG G--------- -------QAT WRLLAVLGGF YIFFLFESFF NLLLPRDQDS
Mouse ZIP6       VFFKFLLSFL VALAVGTLSG DALLHLLPHS HASHQHSHSH EEPAMEMKRG PLFSHLSAQN IEESSYFDST WKGLTALGGL YFMFLVEHVL TLIKQFKDKK
Mouse ZIP14       AFFSRVLTYF IALSIGTLLS NALFQLIPEA FGFNPQDN-- ---------- ---------- --------YV SKSAVVFGGF YLFFFTEKIL KMLLKQKNEH
Consensus                     :. * : .  .::.::                                                         .     :. :          .

                 ....|....| ....|....| ....|....| ....|....| ....|....| ....|....| ....|....| ....|....| ....|....| ....|....|
                        510        520        530        540        550        560        570        580        590        600
Human ZIP12      ---------- ---------- ---------- ---------- ---------- -----QGLSL VNGHVGHSHH LALNSELSDQ AGRGKS---- ----------
Human ZIP1       ---------- ---------- ---------- ---------- ---------- ---------- ---------- QSGPSPLEET R--------- ----------
Yeast ZRT1       ---------- ---------- ---------- ---------- ---------- ----LSHDHT HDEIKDTVVR NTAAVSSEND NENG------ ----------
Arabidopsis ZIP2 ---------- ---------- ---------- ---------- ---------- ---------- ---------- NHVGASVGES R--------- ----------
Mouse ZIP4       EK-------- ---------- ---------- ---------- ---------- -----DGPCS HGGHSHGISL QLAPSNLRQS K--------- ----------
Mouse ZIP6       KKNQKKPEND EDVESKKQLS KYDSQLSSNE EKVDPGERPE SYLRADSQEP SPFDSQQPTM LEEEEVMIAH AHPQEVYNEY VPRGCKNKCH SHFHDTLGQS
Mouse ZIP14       HHGHN----- ---------- ---------- ---------- ---------- -HFTSETLPS KKDQEEGVTE KLQNGDLDHM IPQHCNSELD GKAPGTD---
Consensus                                                                                                   .

                 ....|....| ....|....| ....|....| ....|....| ....|....| ....|....| ....|....| ....|....| ....|....| ....|....|
                        610        620        630        640        650        660        670        680        690        700
Human ZIP12      ---------A STIQLKSPED SQAAEMPIGS MTASNRKCKA ISLLAIMILV GDSLHNFADG LAIGAAFSSS SESGVTTTIA ILCHEIPHEM GD--FAVLLS
Human ZIP1       ---------- -ALLGTVNGG PQHWHDGPGV PQASGAPATP SALRACVLVF SLALHSVFEG LAVGLQRDRA RAMELCLALL LHKGILAVSL SLRLLQSHLR
Yeast ZRT1       ---------T ANGSHDTKNG VEYYEDSDAT SMDVVQSFQA QFYAFLILEF GVIFHSVMIG LNLGS--VGD EFSSLYPVLV FHQSFEGLGI GARLSAIEFP
Arabidopsis ZIP2 ---------- -EDDDVAVKE EGRREIKSGV DVSQALIRTS GFGDTALLIF ALCFHSIFEG IAIGLSDTKS DAWRNLWTIS LHKVFAAVAM GIALLKLIPK
Mouse ZIP4       ---------- -QTHESSRSD LVAEETPELL NPETRRLRAE LRLLPYLITL GDAVHNFADG LAVGAAFSSS WKTGLATSLA VFCHELPHEL GD--FAALLH
Mouse ZIP6       DDLIHHHHDY HHILHHHHHQ NHHPHSHSQR YSREELKDAG IATLAWMVIM GDGLHNFSDG LAIGAAFTEG LSSGLSTSVA VFCHELPHEL GD--FAVLLK
Mouse ZIP14       ---------- EKVIVNSMSV QDLQASQSAC YWLKGVRYSD IGTLAWMITL SDGLHNFIDG LAIGASFTVS VFQGISTSVA ILCEEFPHEL GD--FVILLN
Consensus                                                           :: .  .  .*..  * : :*               :  .        : .

                 ....|....| ....|....| ....|....| ....|....| ....|....| ....|....| ....|....| ....|....| ....|....| ....|....|
                        710        720        730        740        750        760        770        780        790        800
Human ZIP12      SG---LSMKT AILMNFISSL TAFMGLYIGL SVSADPC--- -VQDWIFTVT AGMFLYLSLV EMLPEMTHVQ T-----QRPW MMFLLQNFGL ILGWLSLLLL
Human ZIP1       AQ---VVAGC GILFSCMTPL GIGLGAALAE SAGPLHQL-- -AQSVLEGMA AGTFLYITFL EILPQELASS EQ----RILK VILLLAGFAL LTGLLFIQI-
Yeast ZRT1       RSKRWWPWAL CVAYGLTTPI CVAIGLGVRT RYVSGSYTAL VISGVLDAIS AGILLYTGLV ELLARDFIFN PQR---TKDL RELSFNVICT LFGAGIMALI
Arabidopsis ZIP2 RP---FFLTV VYSFAFGISS PIGVGIGIGI NATSQGAGGD WTYAISMGLA CGVFVYVAVN HLISKGYKPR E-----ECYF DKPIYKFIAV FLGVALLSVV
Mouse ZIP4       AG---LSVKR ALLLNLASAL TAFAGLYVAL AVGVGEE--- -GEAWILAVA TGLFLYVALC DMLPAMMNVR D-----QRPW LLFLLHNVGL LGGWTVLLLL
Mouse ZIP6       AG---MTVKQ AVLYNALSAM LAYLGMATGI FIGHYAEN-- -VSMWIFALT AGLFMYVALV DMVPEMLHND ASDHG-CSRW GYFFLQNAGI LLGFGIMLLI
Mouse ZIP14       AG---MSIQQ ALFFNFLSAC CCYLGLAFGI LAGSHFS--- --ANWIFALA GGMFLYIALA DMFPEMNEVC QEDEKNDSFL VPFVIQNLGL LTGFSIMLVL
Consensus                           .      *                          ::  * ::*  .   :..                     :     . *   :  :

                 ....|....| ...
                        810
Human ZIP12      AIYEQNIKI- ---
Human ZIP1       ---------- ---
Yeast ZRT1       GKWA------ ---
Arabidopsis ZIP2 MIWD------ ---
Mouse ZIP4       SLYEDNITF- ---
Mouse ZIP6       SIFEHKIVFR INF
Mouse ZIP14      TMYSGQIQIG ---
Consensus
```
