# Supplementary material for: Comparative Genomic Analysis of slc39a12/ZIP12: Insight into a Zinc Transporter Required for Vertebrate Nervous System Development
Source: PLoS One. 2014 Nov 6;9(11):e111535. doi: 10.1371/journal.pone.0111535 (PMC4222902; doi:10.1371/journal.pone.0111535)
Supplement: Figure S3 — Sequence alignments of ZIP12 orthologs from different vertebrates. NCBI accession numbers for amino acid sequences are provided in Table 1. The consensus sequence is represented below the alignment, ranging from no (blank), low (.), medium (:), to high conservation (*). (PDF) [file pone.0111535.s003.pdf]

```
                ....|....| ....|....| ....|....| ....|....| ....|....| ....|....| ....|....| ....|....| ....|....| ....|....|
                        10         20         30         40         50         60         70         80         90        100
Human-1         MCFRTKLSVS WVP--LFLLL SRVF--STET DKPSAQDSRS RGSSGQPADL LQ--VLSAGD H-PPHNHSRS LIKTLLEKTG CPRR-RNGMQ GDCNLCFEPD
Human-2         MCFRTKLSVS WVP--LFLLL SRVF--STET DKPSAQDSRS RGSSGQPADL LQ--VLSAGD H-PPHNHSRS LIKTLLEKTG CPRR-RNGMQ GDCNLCFEPD
Chimpanzee-1    MCFRTKLSVS WVP--LFLLL SRVF--STET DKPSAQDSRG RGSSGQLADL LQ--VLSAGD H-PPHNHSRS LIKTLLEKTG CPRR-RNGMR GDCNLCFEPD
Chimpanzee-2    MCFRTKLSVS WVP--LFLLL SRVF--STET DKPSAQDSRG RGSSGQLADL LQ--VLSAGD H-PPHNHSRS LIKTLLEKTG CPRR-RNGMR GDCNLCFEPD
Rhesus monkey   MCFQTKLSIS WVP--LFLLL SRVF--STET DKPSAQDSRG RGSSGQLADL LQ--VLSAGD H-PPHNHSRS LIKTLLEKTG CPRR-RNGMQ GDCHLCFEPD
Galango-1       MCFWTKLSVF WVP--LFLLL SCVT--STET DTPSAQDSNG SGSPGHTADI LR--ALAVGD H-PPLNHSRS LIRTLLEKTG CPQR-RTGMQ GDCDLCFEPD
Galango-2       MCFWTKLSVF WVP--LFLLL SCVT--STET DTPSAQDSNG SGSPGHTADI LR--ALAVGD H-PPLNHSRS LIRTLLEKTG CPQR-RTGMQ GDCDLCFEPD
Rabbit-1        MCFWTKLCVF WMP--LFLPP NPVP--SVEA DKPSTQDGSR TGSPSRRAGW LQ--VLSAGD L-PPLNHSRS LIKTLLEKTG CPRR-TDRMQ GDCNLCLEPD
Rabbit-2        MCFWTKLCVF WMP--LFLPP NPVP--SVEA DKPSTQDGSR TGSPSRRAGW LQ--VLSAGD L-PPLNHSRS LIKTLLEKTG CPRR-TDRMQ GDCNLCLEPD
Guinea pig-1    MCLWTKLSVY GVP--LFLLL SLAS--CTET DKSSTQDSSN PGSPGQLAEI LQ--LLAAGD H-PPLNHSRS LIRTLLEKTG CPR--THGTR GDCHLCFEPD
Guinea pig-2    MCLWTKLSVY GVP--LFLLL SLAS--CTET DKSSTQDSSN PGSPGQLAEI LQ--LLAAGD H-PPLNHSRS LIRTLLEKTG CPR--THGTR GDCHLCFEPD
Mouse           MCFWTNLSVW MIL--LSHSL SLVS--STET GKTLTQNNSR AGSQG-LLEV LR--VLSAGD H-WSLNHPQS LIKILLERTG CPQR-TDWTQ GDCKLCLEAD
Rat             MCFWTELSVW VVL--VSHSF SLAS--STET SKALTQDNSR VGSHS-LLEV LR--VLSAGD D-RSLNHPQS LIKILLERTG CPQR-TDGTQ EDCKLCLEPD
Feral pig-1     MCFWTKLSVF WVP--LFLLL SLFP--STET ENPSTLDSSG PWRPGPLADI LR--VLSAND P-PALNYSRS LIKTLLEKAG CPRR-TYGRQ GHCNLCFEPD
Feral pig-2     MCFWTKLSVF WVP--LFLLL SLFP--STET ENPSTLDSSG PWRPGPLADI LR--VLSAND P-PALNYSRS LIKTLLEKAG CPRR-TYGRQ GHCNLCFEPD
Cow             MCFWTKPSVS WVS--LFLLL SLDP--STET EKPSTQDSSS TWSPGQLTEV LR--VLSADD P-RPLNHSRS LIRTLLEKTG CPRR-TDGRQ GDCNLCFEPD
Horse-1         MCFWTKLSGF WLP--LFLLL SLVP--STET EKPSAQDSSS TWSPGRLTEV LR--IPSAGD P-PPLNHSRS LIRTFLEKTG CPRR-TNGMQ GDCNLCFEPD
Horse-2         MCFWTKLSGF WLP--LFLLL SLVP--STET EKPSAQDSSS TWSPGRLTEV LR--IPSAGD P-PPLNHSRS LIRTFLEKTG CPRR-TNGMQ GDCNLCFEPD
Dog-1           MCFWTKLPVF GVS--LFLLL SLVP--STET ERPSNQDSSS AWSPGHLMEI LR--ALSAGD P-PPRNHSGS LIQTLLEKTG CPRR-TKAMQ GDCSLCFEPD
Dog-2           MCFWTKLPVF GVS--LFLLL SLVP--STET ERPSNQDSSS AWSPGHLMEI LR--ALSAGD P-PPRNHSGS LIQTLLEKTG CPRR-TKAMQ GDCSLCFEPD
Elephant-1      MCFWTKFSIF WMP--LLLLL SLMV--ETQP ENPSTQDNNS TWSPGHLTDV LR--ILSAGD H-PPLNQSKS LMKRLLQETG CPRR-TNGMQ EDCNLCFEPD
Elephant-2      MCFWTKFSIF WMP--LLLLL SLMV--ETQP ENPSTQDNNS TWSPGHLTDV LR--ILSAGD H-PPLNQSKS LMKRLLQETG CPRR-TNGMQ EDCNLCFEPD
Opossum         MHLRTKYFIW GLG--SFFLL PIAAP-EILQ EKPAAQDSIH LQSSGQLMEV LH--ILSNDN YTHTQNYSRR LIQTLLEKTG CPQL-IHGMQ EDCNLCLEPD
Platypus        MCSGKIPGGF WVT--LVFLV SPPARGAQLW RDSPAEGGGS SQGRDRLTQV PRHRQWTYNH TSPLQNRSRG LIRTFMEKTG CPPPGNFGVQ EKCDPCFEPD
Chicken         MCFLSKHPAL GVVCSTFLLS MLIAEGQLG- -EEHGQDGSS APSRGYLMEV LR-LLSADN- PELHTNHSRE LVKMVLERAE CPQR-IYGMQ EDCNLCLEPD
Anole lizard    MSFLTTHLVL LIISLTFLFC LHPARTQQST DKELKVDNSN ASHQSYLADA LR-IISANNY SEHHQNQSRE MIRILLEKTV CQQR-NYEKE DDCNLCLESD
Xenopus frog    MFKWKKSLFW RIP--VFFTF GALF----VC AESRHKDNKQ PPSQGGLLQA LYS-LNSANG TFLHKNNSTD IISLLLEKVE CPKN-VHGKQ DPCTQCLRPD
Tilapia         MHFMSSTLCL LLL---LLYL CPLG------ -RMLEVKGQE QGYLQEALRA LNLPVGPDNK AQLQRNRTSV LITTLLRAVR CTER-TGSCQ DMCEKCLTPD
Consensus       *   .       :                           .                    . .       * .   ::  .:. .  *       .    * *:  .*

                ....|....| ....|....| ....|....| ....|....| ....|....| ....|....| ....|....| ....|....| ....|....| ....|....|
                       110        120        130        140        150        160        170        180        190        200
Human-1         ALLLIAGGNF EDQLREEVVQ RVSLLLLYYI IHQEEICSSK LN-----MSN K-----EYKF YLHSLLSLRQ DEDSSFLSQN ETEDILAFTR QYFD-----T
Human-2         ALLLIAGGNF EDQLREEVVQ RVSLLLLYYI IHQEEICSSK LN-----MSN K-----EYKF YLHSLLSLRQ DEDSSFLSQN ETEDILAFTR QYFD-----T
Chimpanzee-1    ALLLIAGGNF EDQLREEVVQ RVSLLLLYYI IHQEEICSSK LN-----MSN K-----EYKF YLHSLLSLRQ DEDSSFLSQN ETEDILAFTR QYFD-----T
Chimpanzee-2    ALLLIAGGNF EDQLREEVVQ RVSLLLLYYI IHQEEICSSK LN-----MSN K-----EYKF YLHSLLSLRQ DEDSSFLSQN ETEDILAFTR QYFD-----T
Rhesus monkey   ALLLIAGGNF EDQLREEVVQ RVSLLLLYYI IHQEEICSSK LN-----MSN K-----EYKF YLHSLLSLRQ DEDSSFLSQN ETEDILAFTR QYFD-----T
Galango-1       ALLLIAGGNF EDQLQEEVVQ RVSLLLLYYI VHQEEICSSK LN-----MSN K-----EYKF YLHSLLSLRQ DEDSYFLSQN ETEDILSFTR RYFN-----T
Galango-2       ALLLIAGGNF EDQLQEEVVQ RVSLLLLYYI VHQEEICSSK LN-----MSN K-----EYKF YLHSLLSLRQ DEDSYFLSQN ETEDILSFTR RYFN-----T
Rabbit-1        ALLLIAGGGL EDELREEVVQ RVCLLLLYYI IHQQEICASK LN-----MRN K-----EYTF YLHSLLSLRQ NEDSTFLSQN ETEDILAFTR QYFD-----T
Rabbit-2        ALLLIAGGGL EDELREEVVQ RVCLLLLYYI IHQQEICASK LN-----MRN K-----EYTF YLHSLLSLRQ NEDSTFLSQN ETEDILAFTR QYFD-----T
Guinea pig-1    ALILIAGGDL EEQLQEEVIQ RVSLLLLYYI IHQEDICSSK LN-----MSN K-----EYKF YLRSLLSLRQ DEDSYFLSQN ETEDILAFTR QYFD-----T
Guinea pig-2    ALILIAGGDL EEQLQEEVIQ RVSLLLLYYI IHQEDICSSK LN-----MSN K-----EYKF YLRSLLSLRQ DEDSYFLSQN ETEDILAFTR QYFD-----T
Mouse           ALLLTAGGNL EDELREEVVQ RVSLLLLYYI IHQEEICSSK LN-----MSN R-----EYEF YLHSLLGLRQ DEDSYFLSEK ETDDILAFTR KYFG-----T
Rat             ALSLTAGGDL EDELREEVVQ RVSLLLLYYI IHQEEICSSK LN-----MSN R-----EYEF YLHSLLSLRQ DEDSYFLSEK ETDDILASTR KYFG-----T
Feral pig-1     ALLLIAGGGF EDPLGEEVVH RVSLLLLYYI IHQEEICSSK LN-----MSH R-----EYKF YLHSLLSLRQ DEDSYFLSQN EMEDILAFTR QYFD-----T
Feral pig-2     ALLLIAGGGF EDPLGEEVVH RVSLLLLYYI IHQEEICSSK LN-----MSH R-----EYKF YLHSLLSLRQ DEDSYFLSQN EMEDILAFTR QYFD-----T
Cow             ALLLIAGGDL EDQLKEEVIQ RVSLLLLYYI IHQEEICSSK LN-----MSN K-----EYTF YLHSLLSLRQ DEDSYFLSQN ETEDILAFTR QYFD-----T
Horse-1         ALLLIAGGDF EDQLREDVVQ RVSLLLLYYI IHQEEICSSK LN-----MSN K-----EYTF YLHSLLSLRQ DEDSYFLSQN ETEDILAFTR QYFD-----T
Horse-2         ALLLIAGGDF EDQLREDVVQ RVSLLLLYYI IHQEEICSSK LN-----MSN K-----EYTF YLHSLLSLRQ DEDSYFLSQN ETEDILAFTR QYFD-----T
Dog-1           ALLLIAGGDF EEQLQEEVVQ RVSLLLLYYI IHQEEICSSK LN-----MSN K-----EYKF YLHSLLSLRQ DEDSYFLSQN ETEDILALTR RYFD-----T
Dog-2           ALLLIAGGDF EEQLQEEVVQ RVSLLLLYYI IHQEEICSSK LN-----MSN K-----EYKF YLHSLLSLRQ DEDSYFLSQN ETEDILALTR RYFD-----T
Elephant-1      ALLLIAGADF EDQFREEVFQ RISLLLLYYI VHQEEICSSK LS-----MSN T-----EYTF YLHSLLSLRQ DEDSHFLSKN ETEDILAFTR QYFN-----T
Elephant-2      ALLLIAGADF EDQFREEVFQ RISLLLLYYI VHQEEICSSK LS-----MSN T-----EYTF YLHSLLSLRQ DEDSHFLSKN ETEDILAFTR QYFN-----T
Opossum         ALLLIAGGDL EDHLSEEVFQ RISLLLLYYI IHQEEICASK LN-----LSS K-----DYQF YLHSLLSLRQ DEDCYSFSQN ETEDILASVR QHFN-----T
Platypus        TLLLIAGGHV GESPEEEIFQ RISLLLLYYV VHWADVCSPK LV-----LSD K-----EYTF FLHSLLSLRR DENHNFFSRN ETEDALVAVR HHFN-----T
Chicken         AMLLIAGGDL EDHLSEEVFE RISLVLLYYI LHHRDVCSSK LK-----LSN K-----DYKF YLKSMLNLRH DEDCYYFSRN ETEDILAAVK QHFK-----T
Anole lizard    ALILIAGGNL RNYLTEDVFQ KISLILLYYI IHQREACPSM HS-----LRD R-----DYSF FLNSMLN--- -----YFLEN ETSDALEPIR KHFN-----I
Xenopus frog    ALLFMVGINK SSDIDDDAIQ KISVVLLFYI MNQKDFCTSI SN-----LTK M-----DYKF YRNSLLKLRK DEDCHYLSLN EIDDILSVSK QYFQ-----L
Tilapia         IALSVLKDDG KDVLTEEDYQ QISTVLLYYI INLQDLCVSN AAPPPSSFSS SSGNFGNFQF YLLALTNLHP AEDSSFLSSN ETESILQVIN QHYEPSSQHI
Consensus                   .   ::  . ::. :**:*: ::  : * .          :         :: * :  ::            :  : * .. *   . :::

                ....|....| ....|....| ....|....| ....|....| ....|....| ....|....| ....|....| ....|....| ....|....| ....|....|
                       210        220        230        240        250        260        270        280        290        300
Human-1         SQS-QCMETK TLQKKSGIVS SEGANESTLP QLAAMIITLS LQGVCLGQGN LPSPDYFTEY IFSSLNRTNT LRLSELDQLL NTLWTRSTCI KNE-----KI
Human-2         SQS-QCMETK TLQKKSGIVS SEGANESTLP QLAAMIITLS LQGVCLGQGN LPSPDYFTEY IFSSLNRTNT LRLSELDQLL NTLWTRSTCI KNE-----KI
Chimpanzee-1    SQS-QCMETK TLQKKSGIVS SEGANESTLP QLAAMIITLS LQGVCLGQGN LPSPDYFTEY IFSSLNRTNT LRLSELDQLL NTLWTRSTCI KNE-----KI
Chimpanzee-2    SQS-QCMETK TLQKKSGIVS SEGANESTLP QLAAMIITLS LQGVCLGQGN LPSPDYFTEY IFSSLNRTNT LRLSELDQLL NTLWTRSTCI KNE-----KI
Rhesus monkey   SQS-QCMETK TLQKKSGIVS SEGANENTLP QLAATIITLS LQGVCLGQGN LPSPDYFTEY IFSSLNRTHT LRLSELDQLL NTLWTRSTCI KKD-----KI
Galango-1       SRG-QCMETK TLQKKSGILS KHGADENTLP QLAAMIIALS LQGVCLGQRN LPSPDYFTEY IFTFLNSTNT LHLSELDQLL STLWTRSICI KKD-----KI
Galango-2       SRG-QCMETK TLQKKSGILS KHGADENTLP QLAAMIIALS LQGVCLGQRN LPSPDYFTEY IFTFLNSTNT LHLSELDQLL STLWTRSICI KKD-----KI
Rabbit-1        SRS-QCMETE TLQKESGILS SDGADENTLP QLAEMIIALS LQGVCLGRRS LPSPDYFTEY IFSSLNSTHT LHLAELDQLL NILWTKSTCV KKD-----KI
Rabbit-2        SRS-QCMETE TLQKESGILS SDGADENTLP QLAEMIIALS LQGVCLGRRS LPSPDYFTEY IFSSLNSTHT LHLAELDQLL NILWTKSTCV KKD-----KI
Guinea pig-1    SRS-QCMETK MLQKKSGILS SDGADENTLP QLAASIIALS LQGVCLGRRN LPSPDYFTEY IFRFLNSTNN LHLSELDQLL NILWTKSTCN KKD-----EN
Guinea pig-2    SRS-QCMETK MLQKKSGILS SDGADENTLP QLAASIIALS LQGVCLGRRN LPSPDYFTEY IFRFLNSTNN LHLSELDQLL NILWTKSTCN KKD-----EN
Mouse           SSS-QCMETK ILQRESGIQG SNGADEKTLP QLAATIIALS LQGVCLGRKA LPSPDDFTEY IFSFLNSTNT LHLSEIEQLL NMLTTRRTCA KEDK----YL
Rat             STS-PCMETK ILQRESGIQS SNGADEKMLP QLAATIIALS LQGVCLGRKD SPSPDDFTEY IFSFLNRTNA LHLSELEELL NMLSTRRACT KIN-----TL
Feral pig-1     SGS-QCMETK ALQEKSGILS MDGADENTLP QLAATIIALS LQGVCLGQRH LPSPDYFTEY IFSALNSTNT LHLSELDQLL NTLWTRSACI RKD-----KI
Feral pig-2     SGS-QCMETK ALQEKSGILS MDGADENTLP QLAATIIALS LQGVCLGQRH LPSPDYFTEY IFSALNSTNT LHLSELDQLL NTLWTRSACI RKD-----KI
Cow             SRN-QCMETK VLQKKSGILS SDGADENTLP QLAATIIALS LQGVCLGQRN LPPPDYFTEY IFSALNSTNT LHLSELDKLL NTLWTKSTCI RKD-----KI
Horse-1         SRS-KCMETK MLQKKSGILN SHGADEKTLP QLAATIIALS LQGVCLGQRN LPSPDYFMEY IFSSLNSTNT LHLSELDQLL NTLRNKNTCI GKD-----KI
Horse-2         SRS-KCMETK MLQKKSGILN SHGADEKTLP QLAATIIALS LQGVCLGQRN LPSPDYFMEY IFSSLNSTNT LHLSELDQLL NTLRNKNTCI GKD-----KI
Dog-1           SRS-QCMETK TLQEKSGILS SDGADENTLP QLAVTIIALS LQGVCLGQRN LPSPDYFTEY IFSFLNRTTT LHVSELEQLL NTLWTKSTCI RKD-----KI
Dog-2           SRS-QCMETK TLQEKSGILS SDGADENTLP QLAVTIIALS LQGVCLGQRN LPSPDYFTEY IFSFLNRTTT LHVSELEQLL NTLWTKSTCI RKD-----KI
Elephant-1      SRS-QCMETK MLQEKSGILS SKGADENTLP PLAATIVALS LQNVCLGQVN VPSSDYFTEY IFSSLNSTNT LHLSELDQLL NTLWTKSTCY KRD-----KI
Elephant-2      SRS-QCMETK MLQEKSGILS SKGADENTLP PLAATIVALS LQNVCLGQVN VPSSDYFTEY IFSSLNSTNT LHLSELDQLL NTLWTKSTCY KRD-----KI
```

```
Opossum       SRS-QCVETK ILQQKSGIIN NNGADENTLP RLAATIISLS LQGVCLGQRS LPSPDYFMEY IFSSLNSSST LHLPEIDQLL NLLWTKSPCN KRE-----QM
Platypus      SGD-QCLGVD DLEWRSGISR SEGADANTLP QLAMTIITLS LQGVCLGHPR LPSPDFFMDY VFSSLNTTST LRLQELDQLL RLLRAGRPCD HGD-----HT
Chicken       PET-QCVDVS TLEKNADIMD RDGADENTLP RLAALIITLA LQGVCMGKAS LPSPEFFIKY IFNSLNSTTE LQVTELEQLL NVLTARKSCA VNG-----QF
Anole lizard  SET-QCLHVS VLEKEVGILH SNVTDERAVP RLAAAILTFA LRGICLGRRH LPPPEFFMEY IFSFLNSTNE LHA------- ---------- ----------
Xenopus frog  LEEKQCTDVI SMEDAFAMVG STAVDEIKLP RLAEIILTLS LQRVCITRKQ FPNAEFFTDF IFQSFNRTND LYAPDIAELI GELKATGDCS KLSNRRKSVR
Tilapia       PSSSQCIDAA HLLEDADVKG NPGAGTSAVP RVAAAIISHI LQGHCFRKRN LPPPAFFTDY IFHSLNCTSN LQIMDLEELL RQLGVGREGG MHS-------
Consensus          *  .   :     :     ..    :*  :*  *::   *:  *: :    * .  * .: :*  :* :   *

              ....|....| ....|....| ....|....| ....|....| ....|....| ....|....| ....|....| ....|....| ....|....| ....|....|
                     310        320        330        340        350        360        370        380        390        400
Human-1       HQFQRKQNNI ITHDQGYSNF SSS------- ---MEKESED GPVSWDQTCF SARQLVEIFL QKGLSL-ISK EDFKQMSPGI IQQLLS-CSC HLPKD-QQAK
Human-2       HQFQRKQNNI ITHDQDYSNF SSS------- ---MEKESED GPVSWDQTCF SARQLVEIFL QKGLSL-ISK EDFKQMSPGI IQQLLS-CSC HLPKD-QQAK
Chimpanzee-1  HQFQRKQNNI ITRDQDYSNF SSS------- ---MEKESED GPVSWDQTCF SARQLVEIFL QKGLSL-ISK EDFKQMSPGI IQQLLS-CSC HLPKD-QQAK
Chimpanzee-2  HQFQRKQNNI ITRDQDYSNF SSS------- ---MEKESED GPVSWDQTCF SARQLVEIFL QKGLSL-ISK EDFKQMSPGI IQQLLS-CSC HLPKD-QQAK
Rhesus monkey HQLQRKQNSI ITPDQDYSNF SSS------- ---MDEESED GPISWDQTCF SARQLVEIFL QKSLSP-ISK EDFKQMSPGI IQQLLS-CSC HLPKD-QQAK
Galango-1     HQFQRKQNNT TIHDWDYPNL SVS------- ---MDKELDG GPISWDQTCF SARQLVEIFL QNSHPWSISK EDFKQMSPGI IQQLLS-CSC QLPQN-QQAK
Galango-2     HQFQRKQNNT TIHDWDYPNL SVS------- ---MDKELDG GPISWDQTCF SARQLVEIFL QNSHPWSISK EDFKQMSPGI IQQLLS-CSC QLPQN-QQAK
Rabbit-1      HQLQRKQSNI TIHEWGYFNL SAF------- ---MDKESDD GLISWDQTCF SAKQLVEVFL QDSLSP-ISK EDFMQMSPGI IQQLLS-CSC QPPKD-QPTN
Rabbit-2      HQLQRKQSNI TIHEWGYFNL SAF------- ---MDKESDD GLISWDQTCF SAKQLVEVFL QDSLSP-ISK EDFMQMSPGI IQQLLS-CSC QPPKD-QPTN
Guinea pig-1  HQPQRKQDNI KTHDWDYSNL SIS------- ---MDEELDD GPVSWEQTCF SAKQLGEIFL QDSLSP-ISK EDFKHLSPGI IQQLLS-CSC QPSMD-QQA-
Guinea pig-2  HQPQRKQDNI KTHDWDYSNL SIS------- ---MDEELDD GPVSWEQTCF SAKQLGEIFL QDSLSP-ISK EDFKHLSPGI IQQLLS-CSC QPSMD-QQA-
Mouse         HQYQRKQ-NT EEHSLRDPKT STA------- ---MDKESDD HSPSWDQACF SARQLVEIFL QNHSSLSISK EDFKQLSPGI IQQLLS-CSC QMPKD-QQAK
Rat           HEHQRKQ-NT AVHGLRDPKS AAA------- ---MDKVSGD HSVSWDQACF SAQQLVEIFL QNHSSLSISK EDFKQLSPGI IQQLLS-CSC QVPRD-QKAK
Feral pig-1   HQLQRKQNNQ TIHDWDYPNL SIS------- ---VDHESDD GPLSWDQTCF SARQLVEIFL QNRLSP-ISK EDFKQMSPGI IQQLLS-CSC QLPQD-QAAE
Feral pig-2   HQLQRKQNNQ TIHDWDYPNL SIS------- ---VDHESDD GPLSWDQTCF SARQLVEIFL QNRLSP-ISK EDFKQMSPGI IQQLLS-CSC QLPQD-QAAE
Cow           HQLRRKQNSL TLHDGDYSNL SVS------- ---MDPESED GPISWDQTCF SAGQLVNIFL QNKLSP-ISK EDFKQMSPGI IQQLLS-CSC QLPQN-QQAK
Horse-1       HQLKRKQNNI TIHDWGYPNL SVS------- ---MNQESDD GPVSWDQTCF SAGQLVEIFL QNSLSP-ISK EDFKQMSPGI IQQLLS-CSC QLPKD-QQAK
Horse-2       HQLKRKQNNI TIHDWGYPNL SVS------- ---MNQESDD GPVSWDQTCF SAGQLVEIFL QNSLSP-ISK EDFKQMSPGI IQQLLS-CSC QLPKD-QQAK
Dog-1         HQLQRKQNNL TIYDWG-SNL SVS------- ---MDQESHD GSISWDESCF SAGQLVEIFL QNSLSP-ISK EDFKRMSPGI IQQLLS-CSC QLPKD-QQAK
Dog-2         HQLQRKQNNL TIYDWG-SNL SVS------- ---MDQESHD GSISWDESCF SAGQLVEIFL QNSLSP-ISK EDFKRMSPGI IQQLLS-CSC QLPKD-QQAK
Elephant-1    YQLQRKQNNI PTLDRGYPNL SVS------- ---VDKESEV GPFSWDQTCF TARQLVEIFS QNSRLP-ISK EVFKQMSPAI LQQLLS-CSC QLPKD-PQAK
Elephant-2    YQLQRKQNNI PTLDRGYPNL SVS------- ---VDKESEV GPFSWDQTCF TARQLVEIFS QNSRLP-ISK EVFKQMSPAI LQQLLS-CSC QLPKD-PQAK
Opossum       FPHKRRWRNL TIQDWTHLNL SASA------ --TLDGDSND NSAALNQACF SAKELVEIFL PNSHSP-ISK EHFQQISPAI IQQLLS-CSC QLTKY-QQAK
Platypus      QGFPRKKKLI VGRECAYLLF SNSLPAGE-- --LSAAESES FHLLLHQACL SAEDLAEIFL PASQGS-ISK ENFQEISPAV IQQLLLTCPC GSDDP-QLVR
Chicken       QSYKRRHYGM TGTGTGARNI PIKANYTEED YLKAYYKDRG SNTNWDQVCF SADELVNIFL KNSSSS-ISK EHFKQMSPAI VQQLLS-CSC QLPES-KQTR
Anole lizard  ---------- ---------- ---------- ---------- --------CF SASELVEIFL HDSHLS-VSK EHFRQMSPAI IQQLLS-CSC KLIES-KQAK
Xenopus frog  SVIPQKDNEL LSNTNEEKGH DHS------- -DIYEVEAHS RYWHQTHSCF SVYELVKVFF KNSHSP-VSK EQFKQISPAI IHQLLT-CSC PPASNGTTRL
Tilapia       -RNRKRRGIR VGSKKVARHL QDS------- ---CHPEIEG INSDWAQVCF SANQLVDIFA LDPHLP-ISK EHFRQICPAI IQQLLG-NAC EPEEQ-QTRG
Consensus                                                         *: :. :* .:*         :** * * .:.*.: ::***   .*

              ....|....| ....|....| ....|....| ....|....| ....|....| ....|....| ....|....| ....|....| ....|....| ....|....|
                     410        420        430        440        450        460        470        480        490        500
Human-1       LPPTTLEKYG YSTVAVTLLT LGSMLGTALV LFHSCEENYR LILQLFVGLA VGTLSGDALL HLIPQVLGLH K-----QEAP EFGHFHESKG HIWKLMGLIG
Human-2       LPPTTLEKYG YSTVAVTLLT LGSMLGTALV LFHSCEENYR LILQLFVGLA VGTLSGDALL HLIPQVLGLH K-----QEAP EFGHFHESKG HIWKLMGLIG
Chimpanzee-1  LPPTTLEKYG YSTVAVTLLT LGSMLGTALV LFHSCEENYR LILQLFVGLA VGTLSGDALL HLIPQVLGLH K-----QEAP EFGHFHESKG HIWKLLGLIG
Chimpanzee-2  LPPTTLEKYG YSTVAVTLLT LGSMLGTALV LFHSCEENYR LILQLFVGLA VGTLSGDALL HLIPQVLGLH K-----QEAP EFGHFHESKG HIWKLLGLIG
Rhesus monkey LPPTTLEKYG YSTVAVTLLT LGSMLGTALV LFHSCEENYR LILQLFVGLA VGTLSGDALL HLIPQVLGLH K-----QEAP EFGHFHESKG HIWKLLGLIG
Galango-1     LPPSTLEKYG YSTVAVTVLT LGSMLGTALV LFHSCEENYK LILQLFVGLA VGTLSGDALL HLIPQILGLH K-----QEAP EFEHFHESQG HIWKLLGLIG
Galango-2     LPPSTLEKYG YSTVAVTVLT LGSMLGTALV LFHSCEENYK LILQLFVGLA VGTLSGDALL HLIPQILGLH K-----QEAP EFEHFHESQG HIWKLLGLIG
Rabbit-1      LPPSTLEKYG YSTVAVALLT LGSMLGTALV LFHGCEENYS LVLQLFVGLA VGTLSGDALL HLIPQVLGLH K-----QEAP GFGHVHESKG HIWKLLGLIG
Rabbit-2      LPPSTLEKYG YSTVAVALLT LGSMLGTALV LFHGCEENYS LVLQLFVGLA VGTLSGDALL HLIPQVLGLH K-----QEAP GFGHVHESKG HIWKLLGLIG
Guinea pig-1  LPPTTLEKYG YSTGAVMLLT LGSMLGTALV LFHSCEENYS LILQLFVGLA VGTLSGDALL HLIPQVLDLH R-----QESL EFGHFHESKG HIWKLLGLIG
Guinea pig-2  LPPTTLEKYG YSTGAVMLLT LGSMLGTALV LFHSCEENYS LILQLFVGLA VGTLSGDALL HLIPQVLDLH R-----QESL EFGHFHESKG HIWKLLGLIG
Mouse         PPPTTLEKYG YSTVAVTLLT LGSMLGTALV LFHSCEENYS LILQLFVGLA VGTLSGDALL HLIPQVLGLH K-----QEA- EFGHFHESQS PIWKLLGLLG
Rat           PPPTTLEKYG YSTVAVTLLT LGSMLGTALV LFHSCEENYS LILQLFVGLA VGTLSGDALL HLIPQVLGLH K-----QEA- ELGHFHESQS PIWKLLGLLG
Feral pig-1   LSPTTLEKYG YSTVAVTLLT LGSMLGTTLI LFHSCEENYR LILQLFVGLA VGTLSGDALL HLIPQILDVH K-----QEAL EFGHFHESKG YIWKLLALIG
Feral pig-2   LSPTTLEKYG YSTVAVTLLT LGSMLGTTLI LFHSCEENYR LILQLFVGLA VGTLSGDALL HLIPQILDVH K-----QEAL EFGHFHESKG YIWKLLALIG
Cow           IAPTTLEKYG YSTVAVTLLT LGSMLGTTLI LFHSCEENYR LILQLFVGLA VGTLSGDALL HLIPQILGLH M-----QETS EFGHFYENKG HIWKLLGLIG
Horse-1       PPPTTLEKYG YSTVAVTLLT LGSMLGTTLI LFHSCEENYR LILQLFVGLA VGTLSGDALL HLIPQVLGLH K-----QEAS EFGHFHESKG HIWQLLGLIG
Horse-2       PPPTTLEKYG YSTVAVTLLT LGSMLGTTLI LFHSCEENYR LILQLFVGLA VGTLSGDALL HLIPQVLGLH K-----QEAS EFGHFHESKG HIWQLLGLIG
Dog-1         LPPTTLEKYG YSTVAVALLT LGSMLGTTLI LFHSCEN-YR LILQLFVGLA VGTLTGDALL HLLPQVLGLH K-----QEGS EFGHFHESKG HIWKLLGLIG
Dog-2         LPPTTLEKYG YSTVAVALLT LGSMLGTTLI LFHSCEN-YR LILQLFVGLA VGTLTGDALL HLLPQVLGLH K-----QEGS EFGHFHESKG HIWKLLGLIG
Elephant-1    PPPTTLEKYG YSTVAVTLIT LGSMLGTTLI LFSSCEENYR LILQLFVGLA VGTLSGDALL HLIPQILGLH K-----QEAS GFGHFHESKG HIWKLLGLIG
Elephant-2    PPPTTLEKYG YSTVAVTLIT LGSMLGTTLI LFSSCEENYR LILQLFVGLA VGTLSGDALL HLIPQILGLH K-----QEAS GFGHFHESKG HIWKLLGLIG
Opossum       LPPTALEKYG YSTAAVLLIT VGSMFGTTLI LFNNCEENYK LVLQLFVGLA VGTLSGDALL HLIPQILGLH K-----QEAQ ELGHVHEGKE YIWKLLGLIG
Platypus      TPPEALERYG YGTVAVLLIT MGSVLGPGLI LLSGCYGSSQ LVMQLFVGLA IGVLSGDALL HLIPQILGLH K-----QEAQ KMGHFHEGKE YIWKLLGVIG
Chicken       LPPTTLEKYG YSTVAVLLIT VGSMFGTSLI LFNSCQEIYT LILQLFVGLA VGTLSGDALL HLIPQTLGLH K-----HEAQ EVEHFYEGKE YIWKLLGIIG
Anole lizard  PQPKSLEKYG YSTLAVLLIT VGSMFGTTLI LFNSCQECYT LILQLFVGLA VGTLSGDALL HLIPQVLGLH T-----HSIQ KNGKFYEGKE HIWKMLALIG
Xenopus frog  APPTTLEKYG YSTISVLLLT IGSMFGATLI IFSSCEENYK LILQLFVGLA VGTLSGDALL HLIPQVLGVH EVVTHEHHMQ QGDYIFEENE YLWKIMGMIG
Tilapia       SLPTALEKYG YSTAAVFLIT VGSMFGICLI FFNSCQETYS LILQLFVGLA VGTLSGDALL HLIPQILGLH HS----THNH GDEDYDEEKQ YLWKILGVIA
Consensus       * :**:** *.* :* ::* :**::*  *: :: .*      *::******* :*.*:***** **:** *.:*                  * :   :*:::.::.

              ....|....| ....|....| ....|....| ....|....| ....|....| ....|....| ....|....| ....|....| ....|....| ....|....|
                     510        520        530        540        550        560        570        580        590        600
Human-1       GIHGFFLIEK CFILLVSPND K--------- ---------- ---------- --------KS PEDSQAAEMP IGS----MTA SNRKCKAISL LAIMILVGDS
Human-2       GIHGFFLIEK CFILLVSPND KQGLSLVNGH VGHSHHLALN SELSDQAGRG KSASTIQLKS PEDSQAAEMP IGS----MTA SNRKCKAISL LAIMILVGDS
Chimpanzee-1  GIHGFFLIEK CFILLVSPND K--------- ---------- ---------- --------KS PEDSQAAEMP IGS----MTA SNRKCKAISL LAIMILVGDS
Chimpanzee-2  GIHGFFLIEK CFILLVSPND KQGLSLVNGH VGHSHHLALN SELSDQAGRG KSASTIQLKS PEDSQAAEMP IGS----MTA SNRKCKAISL LAIMILVGDS
Rhesus monkey GIHGFFLIEK CFILLVSPND KQGLSLVNGH VGHSHHLALN SELSDQAGRG KSASTIQLKS PEDSQAAEMP IGS----MTA PNRKCKVISL LAIMILVGDS
Galango-1     GIHGFFLLEK CFVLLVSPND KG-LSLVNGH MGHSHHLELN SELSDQADRG KSSSTMQLKG PEDSQTAEIP IGS----VTS SGSKCKAISL LAIMILVGDS
Galango-2     GIHGFFLLEK CFVLLVSPND K--------- ---------- ---------- --------KG PEDSQTAEIP IGS----VTS SGSKCKAISL LAIMILVGDS
Rabbit-1      GIHGFFLIEK CFILLVSPNA KQGLSPVNGH VGHSHHVTLN SELSDQSGRG KSASMIQLKG PEDSQAAEIP ISS----MTA SNRKCKAISL LAVMILVGDS
Rabbit-2      GIHGFFLIEK CFILLVSPNA KK-------- ---------- ---------- ---------G PEDSQAAEIP ISS----MTA SNRKCKAISL LAVMILVGDS
Guinea pig-1  GIHGFFLIEK CFILLVSPNA KG-LSLINGH VGHPHHPALN PELDDQSGRG KSASTIQLKG PEDSQAAEIS VAS----MTT SNRKCKTISL LAVMILVGDS
Guinea pig-2  GIHGFFLIEK CFILLVSPNA K--------- ---------- ---------- --------KG PEDSQAAEIS VAS----MTT SNRKCKTISL LAVMILVGDS
Mouse         GIHGFFLIEK CFILLVSPNT KG-LPLVNEH VGHTHHLGLN PELNDQSSGG KSISTIQLKG PEDSQTTVLP IGN----VPA SNRNGKTISL LAIMILVGDS
Rat           GIHGFFLIEK CFILLVSPNT KG-LPLVNGH AGHTHHLGLS PELNDQSGGG KSISTIQLKG PEDSQTAELP KGN----VPA SNRNRKTISL LAVMVLVGDG
Feral pig-1   GIHGFFLIEK CFILLVSPSA EQGVSLVTGH AGHTHHLAFN SELSDQTGRG KSASTIQLKS PEDSQAAEIP RGS----MTA PNRKCKTISL LAIMILVGDS
```

```
Feral pig-2   GIHGFFLIEK CFILLVSPSA EK-------- ---------- ---------- ---------S PEDSQAAEIP RGS----MTA PNRKCKTISL LAIMILVGDS
Cow           GIHGFFLIEK CFTLLVSPGA KK-------- ---------- ---------- ---------G PEDAQAAEIP IPS----VNT PNRKCKTISL LAIMILVGDS
Horse-1       GIHGFFLIEK CFILLVSPGA KQGVSLVNGH VGHSHHLALN SELSDQSGRG KSASTIPLKG PEDSQAPEIH IGS----MTA SNRKCETISL LAVMILVGDS
Horse-2       GIHGFFLIEK CFILLVSPGA KK-------- ---------- ---------- ---------G PEDSQAPEIH IGS----MTA SNRKCETISL LAVMILVGDS
Dog-1         GIHGFFLIEK CFILLVSPNA EG-VSLVNGH GGHSHHLVLN SELSDQSGRG KSASTLQLKS PEDSQAAEIP GGS----MTA SNRKCNTISL LAIMILVGDS
Dog-2         GIHGFFLIEK CFILLVSPNA E--------- ---------- ---------- --------KS PEDSQAAEIP GGS----MTA SNRKCNTISL LAIMILVGDS
Elephant-1    GIHGFFLIEK CFILLVSPNA KQDASLVNGH VGHSHHFALD SELSDQSGGG QSTSTIQLKS PEDSQAAEIP IRR----KTA FNRKCNTMSL LAIMILVGDS
Elephant-2    GIHGFFLIEK CFILLVSPNA K--------- ---------- ---------- --------KS PEDSQAAEIP IRR----KTA FNRKCNTMSL LAIMILVGDS
Opossum       GIHGFFLIEK CFLLFISPNA KK-------- ---------- ---------- ---------G PEDSDTAEIP IDS----LAA SNRKRKAISL LAIMILVGDS
Platypus      GIHGFFLIEK CFVLLMPESD KR-------- ---------- ---------- ---------S PEDSEPVRDS ADS----LTA SKGKRKAIGR LAIMVLLGDS
Chicken       GIHGFFLIEK CFFLLVTPRE QR-------- ---------- ---------- ---------S PEDSESAEVP PGN-----KV ISTKNKRISL LAIMVLVGDS
Anole lizard  GIYGFFLIEK GFVLFMSPHE HV-------- ---------- ---------- ---------S LAILMNTNIS EAHYLSLFNK LSFRCQGIGL LAIMILVGDS
Xenopus frog  GIYGFFLINK LFFLLVSSPK QG-LSLMNGH LTHSHDLPLD AHLGDQSRKA KSSSTIQLRS PDDCEHSEVP ETLP--VRPK ARRKSKGISL LAIMILVGDS
Tilapia       GIYSFFLIEK LFSFLVPSHS HG-------- ----HGGDLS LNCNGHSQRG KSISTIQLGP VDDLECTEMS PEHAN--TRR PSHPRQGVSL LAVMVIVGDS
Consensus     **:.***::*  * :::.    .                                                                      : :.  **:*:::**.

              ....|....| ....|....| ....|....| ....|....| ....|....| ....|....| ....|....| ....|....| ....|....| ....|....|
                     610        620        630        640        650        660        670        680        690        700
Human-1       LHNFADGLAI GAAFSSSSES GVTTTIAILC HEIPHEMGDF AVLLSSGLSM KTAILMNFIS SLTAFMGLYI GLSVSADPCV QDWIFTVTAG MFLYLSLVEM
Human-2       LHNFADGLAI GAAFSSSSES GVTTTIAILC HEIPHEMGDF AVLLSSGLSM KTAILMNFIS SLTAFMGLYI GLSVSADPCV QDWIFTVTAG MFLYLSLVEM
Chimpanzee-1  LHNFADGLAI GAAFSSSSES GVTTTIAILC HEIPHEMGDF AVLLSSGLSM KTAILMNFIS SLTAFMGLYI GLSVSADPCV QDWIFTVTAG MFLYLSLVEM
Chimpanzee-2  LHNFADGLAI GAAFSSSSES GVTTTIAILC HEIPHEMGDF AVLLSSGLSM KTAILMNFIS SLTAFMGLYI GLSVSADPCV QDWIFTVTAG MFLYLSLVEM
Rhesus monkey LHNFADGLAI GAAFSSSSES GVTTTIAILC HEIPHEMGDF AVLLSSGLSM KTAILMNFIS SLTAFIGLYI GLSVSADPCV QDWIFTVTAG MFLYLSLVEM
Galango-1     LHNFADGLVI GAAFSASSES GVTTTIAILC HEIPHEMGDF AVLLSSGLSV KTAILMNFIS ALTAFIGLYI GLSVSADPGV QDWIFTVTAG MFLYLSLVEM
Galango-2     LHNFADGLVI GAAFSASSES GVTTTIAILC HEIPHEMGDF AVLLSSGLSV KTAILMNFIS ALTAFIGLYI GLSVSADPGV QDWIFTVTAG MFLYLSLVEM
Rabbit-1      LHNFADGLVI GAAFSSSCES GVTTTIAILC HEIPHEMGDF AVLLSSGLPV KTAILMNFVS ALTAFLGLYI GLSVSADPCV QDWILTVTAG MFLYLSLVEM
Rabbit-2      LHNFADGLVI GAAFSSSCES GVTTTIAILC HEIPHEMGDF AVLLSSGLPV KTAILMNFVS ALTAFLGLYI GLSVSADPCV QDWILTVTAG MFLYLSLVEM
Guinea pig-1  LHNFADGLVL GAAFSSSSES GVTTTIAILC HEIPHEMGDF AVLLSSGLSV KTAILMNFIS ALTAFIGLYI GLSVSADPCV QDWILTVTAG MFLYLSLVEM
Guinea pig-2  LHNFADGLVL GAAFSSSSES GVTTTIAILC HEIPHEMGDF AVLLSSGLSV KTAILMNFIS ALTAFIGLYI GLSVSADPCV QDWILTVTAG MFLYLSLVEM
Mouse         LHNFADGLVI GTAFSSSLES GVTTTIAILC HEIPHEMGDF AVLLSSGLSV RTAILMNFLS ALTAFAGLYI GLSVSADPRV QDWILTVTAG MFLYLSLVGM
Rat           LHNFADGLVI GTAFSSSLES GVTTTIAILC HEIPHEMGDF AVLLSSGLSI RTAILMNFLS ALTAFIGLYI GLSVSADPRV QDWILTVTAG MFLYLSLVGM
Feral pig-1   LHNFADGLVI GAAFSSSSES GVTTTIAILC HEIPHEMGDF AVLLGSGLPI KIAILVNFIS ALTAFVGLYI GLAVSTDPCI QNWILTVTAG MFLYLSLVEM
Feral pig-2   LHNFADGLVI GAAFSSSSES GVTTTIAILC HEIPHEMGDF AVLLGSGLPI KIAILVNFIS ALTAFVGLYI GLAVSTDPCI QNWILTVTAG MFLYLSLVEM
Cow           LHNFADGLVI GAAFSSSSEA GVTTTIAILC HEIPHEMGDF AVLLSSGLPV KIAILMNFIS ALTAFIGLYI GLSVSTDPCI QNWILTVTAG MFLYLSLVEM
Horse-1       LHNFADGLVI GAAFSSSSES GVTTTIAILC HEIPHEMGDF AVLLSSGLPV KIAILMNFIS ALTAFIGLYI GLSVSTDPCV QNWILTITAG MFLYLSLVEM
Horse-2       LHNFADGLVI GAAFSSSSES GVTTTIAILC HEIPHEMGDF AVLLSSGLPV KIAILMNFIS ALTAFIGLYI GLSVSTDPCV QNWILTITAG MFLYLSLVEM
Dog-1         LHNFADGLVI GAAFSSSSES GATTTLAILC HEIPHEMGDF AVLLSSGIPI KIAILMNFLS ALTAFIGLYV GLSVSTDPCV QNWILTVTAG MFLYLSLVEM
Dog-2         LHNFADGLVI GAAFSSSSES GATTTLAILC HEIPHEMGDF AVLLSSGIPI KIAILMNFLS ALTAFIGLYV GLSVSTDPCV QNWILTVTAG MFLYLSLVEM
Elephant-1    LHNFADGLVI GAAFSSSSES GITTTIAILC HEIPHEMGDF AVLLSSGLSV KIAILVNFMS ALTAFIGLYI GLSVSTDPCV QNWILTVTAG MFLYLSLVEM
Elephant-2    LHNFADGLVI GAAFSSSSES GITTTIAILC HEIPHEMGDF AVLLSSGLSV KIAILVNFMS ALTAFIGLYI GLSVSTDPCV QNWILTVTAG MFLYLSLVEM
Opossum       LHNFADGLVI GAAFSSSTES GVTTTIAILC HEIPHEMGDF AVLLSSGLAI KIAILMNFIS ALTAFIGLYI GLSVSTDPCI QNWILTVTAG MFLYLSLVEM
Platypus      LHNFADGLVI GAAFSSSTET GVTTAVAILC HEIPHEMGDF AVLLSTGMLT KTAVLINFLS ALPAFLGLYV GLAISADPGV QNWIFSVTAG MFLYLSLVEM
Chicken       LHNFADGLVI GAAFSSSTET GVTTTIAILC HEIPHEMGDF AVLLSTGLPT KIAIFMNFIS ALTAFLGLYI GLSVSTDPCI QNWIFTVTAG MFLYLSLAEM
Anole lizard  LHNFADGLVI GAAFSSSTEM GATTTIAILC HEIPHEMGDF AVLLSTGLSS KIAILMNFIS ALTAFIGLYI GLSISTDPCI QNWIFTVTAG MFLYLSLVEM
Xenopus frog  LHNFADGMVI GSAFSSSTET GIASTVAILC HEIPHEMGDF AVLLNTGLSA KIAFLMNFIS ALTAFAGLYI GLSLSANPDV QIWIFAVTAG MFLYLSLVEM
Tilapia       LHNFADGLVI GAAFSSSSET GMATTVAILC HEIPHEMGDF AVLLSSGLSV KTAVLMNFLS ALTAFMGLYI GLFVSSETEV QQWIFSVTAG IFLYLSLVEM
Consensus     *******:.: *:***:* *   * :::**** ********** ****.:*:   : *.::**:* :*.** ***: ** :*::. : * **:::*** :******. *

              ....|....| ....|....| ....|....| ....|....| ..
                     710        720        730        740
Human-1       LPEMTHVQTQ RPWMMFLLQN FGLILGWLSL LLLAIYEQNI KI
Human-2       LPEMTHVQTQ RPWMMFLLQN FGLILGWLSL LLLAIYEQNI KI
Chimpanzee-1  LPEMTHVQTQ RPWMMFLLQN FGLILGWLSL LLLAIYEQNI KI
Chimpanzee-2  LPEMTHVQTQ RPWMMFLLQN FGLILGWLSL LLLAIYEQNI KI
Rhesus monkey LPEMTHVQTQ RPWMMFLLQN FGLILGWLSL LLLAIYEQNI KI
Galango-1     LPEMTHVQTQ RPWMMFLLQN FGLTLGWLSL LLLAIYEQNI KI
Galango-2     LPEMTHVQTQ RPWMMFLLQN FGLTLGWLSL LLLAIYEQNI KI
Rabbit-1      LPEMTHVQTR RPWMMFLFQN LGLILGWLSL FLLAIYEQNI KI
Rabbit-2      LPEMTHVQTR RPWMMFLFQN LGLILGWLSL FLLAIYEQNI KI
Guinea pig-1  LPEMTHVQTR RPWMMFLLQN IGLILGWLSL LLLAIYEQNI KI
Guinea pig-2  LPEMTHVQTR RPWMMFLLQN IGLILGWLSL LLLAIYEQNI KI
Mouse         LPEMTHVQTQ RPWMTFLLQN VGLVLGWFSL LLLAVYEQNI KI
Rat           LPEMTHVQTQ RPWMTFLLQN VGLVLGWFSL LLLAVYEQNI KI
Feral pig-1   LPEMTHVQTR RPWLMFLLQN FGLTLGWLSL LLLAIYEQNI KI
Feral pig-2   LPEMTHVQTR RPWLMFLLQN FGLTLGWLSL LLLAIYEQNI KI
Cow           LPEMTHVQTQ RPWLMFLLQN FGLTLGWLSL FLLAIYEQNI KI
Horse-1       LPEMTHVQTR RPWLMFLLQN FGLTLGWLSL LLLAIYEQNI KI
Horse-2       LPEMTHVQTR RPWLMFLLQN FGLTLGWLSL LLLAIYEQNI KI
Dog-1         LPEMTHVQTR RPWLMFLLQN FGLTLGWLSL LLLAIYEQNI KI
Dog-2         LPEMTHVQTR RPWLMFLLQN FGLTLGWLSL LLLAIYEQNI KI
Elephant-1    LPEMTHVQAR RPWLMFLLQN LGLILGWLFL LLLAIYEDNI KI
Elephant-2    LPEMTHVQAR RPWLMFLLQN LGLILGWLFL LLLAIYEDNI KI
Opossum       LPEMTHVQTQ RPWLMFLLQN FGLILGWLSL LLLAIYEHNI KI
Platypus      LPEMTHVQTQ RPWLMFLLQN FGLILGWLLL LLLAIYEHNI KI
Chicken       LPEMTHIQTR RPWLMFLLQN LGLLLGWLCL LLLAIYEQKI KI
Anole lizard  LPEMTHIQTQ RPWLMFLLQN LGLLLGWLCL LLLAIYEPKI KI
Xenopus frog  LPEMIHVQTQ RPWLMFFLQN LGLLLGWLCL LLLAIYEHKI QF
Tilapia       LPEMNRVKTD RPCLMFFLQN LGLLLGWACL LLLALFEHEL KF
Consensus     **** ::::  ** : *::** .** ***  * :***::* :: ::
```
